# Predictive modeling of ADME properties using M-polynomial based topological indices for biocompatible polysaccharides

**DOI:** 10.1038/s41598-025-14134-5

**Published:** 2025-08-13

**Authors:** W. Eltayeb Ahmed, Muhammad Naeem, Muhammad Kamran Siddiqui, Mohamed Abubakar Fiidow

**Affiliations:** 1 Department of Mathematics and Statistics, College of Science, Imam Mohammad Ibn Saud Islamic University (IMSIU), Riyadh, Saudi Arabia; 2 Department of Mathematics, National University of Sciences and Technology (NUST), Islamabad, Pakistan; 3 Department of Mathematics, Comsats University Islamabad, Lahore Campus, Islamabad, Pakistan; 4 Department of Mathematical Sciences, Faculty of Science, Somali National University, Mogadishu Campus, Mogadishu, Somalia

**Keywords:** ADME properties, Statistical analysis, Polycyclic drug molecules, M-polynomial indices, Physical chemistry, Applied mathematics, Information technology, Software, Statistics

## Abstract

Dextran and chitosan, two natural polysaccharides, are recognized for their biocompatibility, biodegradability, and structural adaptability. Dextran, composed of glucose units with predominant $\alpha$-(1$\rightarrow$6) linkages, exhibits flexible conformations influenced by branching and molecular weight. Chitosan, derived from chitin via deacetylation, consists of $\beta$-(1$\rightarrow$4)-linked D-glucosamine units and displays semi-crystalline behavior sensitive to pH and ionic conditions. An in-depth understanding of these structural properties is essential for applications in drug delivery, biomedical engineering, and polymer-based therapeutics. In this study, M-polynomial indices were calculated for dextran and chitosan using the edge/connectivity partition technique. Their predictive utility was evaluated through statistical correlations with several ADME-related physico-chemical properties of polycyclic drugs. Multiple regression models−Support Vector Regression, Lasso, Ridge, ElasticNet, and Multiple Linear Regression−were applied to model these relationships. Performance assessment was conducted using cross-validation and external test metrics, including the coefficient of determination ($R^2$), Pearson correlation coefficient (R), root mean squared error, and *p*-values. Findings indicate that M-polynomial indices can reliably predict key properties such as molecular weight, exact mass, molar refractivity, polarization, complexity, and others. Several models demonstrated excellent predictive strength (e.g., $R^2 > 0.95$) with statistical significance ($p < 0.001$), confirmed through both cross-validation and external validation. A Python-based tool was also developed to automate the computation of M-polynomial indices, enhancing efficiency and reproducibility. The results support the biological relevance of topological descriptors in modeling drug behavior and underline their potential utility in computational drug design, especially for biocompatible polysaccharide-based delivery systems.

## Introduction

   The results of small-molecule drug research are difficult and challenging. The traditional method of small molecule drug development usually puts a molecule’s effectiveness ahead of its drugability, which commonly leads to higher failure rates and research costs. Reports indicate that obtaining a single approved medication required around 30.4 preclinical new chemical entities (NCEs) between 2007 and 2011, compared to just 12.4 NCEs needed between 2003 and 2007^1^. Therefore, the increased failure rates in clinical and preclinical studies might be linked to the rising costs of new drug development and research. Effective measures to improve the ratio of pharmaceutical research and development are therefore crucial.

The molecular structure of a medication dictates its physicochemical qualities, which subsequently influence its absorption, distribution, metabolism, and excretion (ADME) characteristics. Pharmaceutical chemistry can modulate the pharmacological action of drug compounds by structural modifications. Approved medications have successfully undergone rigorous pre-clinical and clinical trials, hence confirming their favourable drug-like qualities. Summarising the characteristics of the structures of globally small molecular drugs as a principle for selecting, designing, and optimizing lead compounds and drug applicants in the initial phases of preclinical research would enhance the successive ratio of drug development, while also preemptively eliminating suboptimal drug-like compounds and reducing research and development costs. Consequently, the examination of the essential chemical structural characteristics of small molecule pharmaceuticals holds considerable theoretical and practical significance.

Sugiyama defines drug-like features as the physicochemical and biological characteristics (ADME) that align with optimal clinical efficacy^2^. Lipinski et al.^3^ fins that the term drug-like chemical denotes those compounds possessing acceptable ADME qualities and capable of withstanding Phase I clinical trials. Borchardt emphasized that the duty of medicinal chemists extends beyond enhancing the pharmacological efficacy of therapeutic compounds to include improving their drug-like characteristics^4^. Consequently, an organic compound sanctioned for a disease must exhibit sufficient pharmacological efficacy and drug-like characteristics. Since the introduction of Lipinski “rule of five” in 1997, researchers have intensified their attention on the drug-like property criteria of lead compounds^5^. The physicochemical characteristics examined in these studies^6–11^.

Research on drug-like features seeks to assist researchers in designing molecules with favourable ADME characteristics during the first stages of drug discovery, hence potentially decreasing the failure rate and expenses associated with drug development. To attain this objective, medicinal chemists have extensively researched methods to enhance the drug-like characteristics of organic compounds. The substitution of a non-ionogenic group with an ionogenic group enhances the permeability of an organic molecule, hence influencing its in vivo oral bioavailability^12^. Reducing hydrogen bonds and augmenting lipophilicity will improve an organic molecule’s capacity to traverse the blood-brain barrier^13^.

Since 2001, when van de Waterbeemd et al. introduced the notion of “property-based design,” there has been heightened emphasis on its use in drug discovery^14^. The correlation between structure and physicochemical qualities has garnered the interest of chemists as a novel technique in drug development, complementing structure-activity correlations in the advancement of drug discovery.

**Fig. 1:**
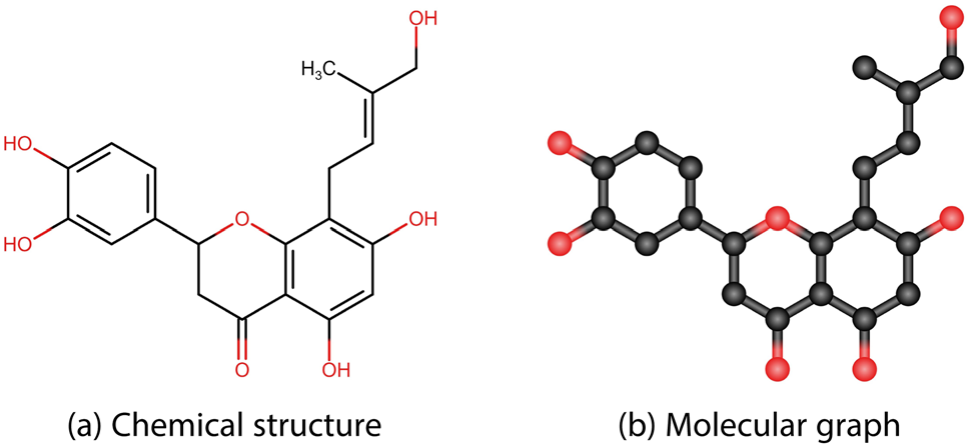
Chemical and molecular graph of polycyclic structured drugs.

**Fig. 2:**
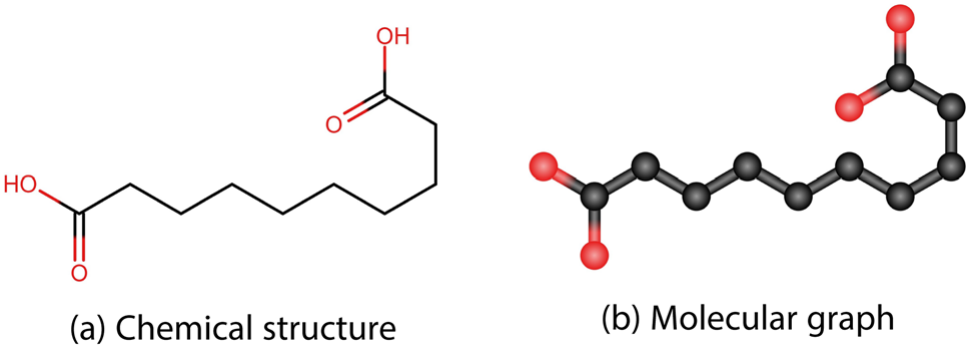
Chemical and molecular graph of acyclic structured drugs.

**Fig. 3:**
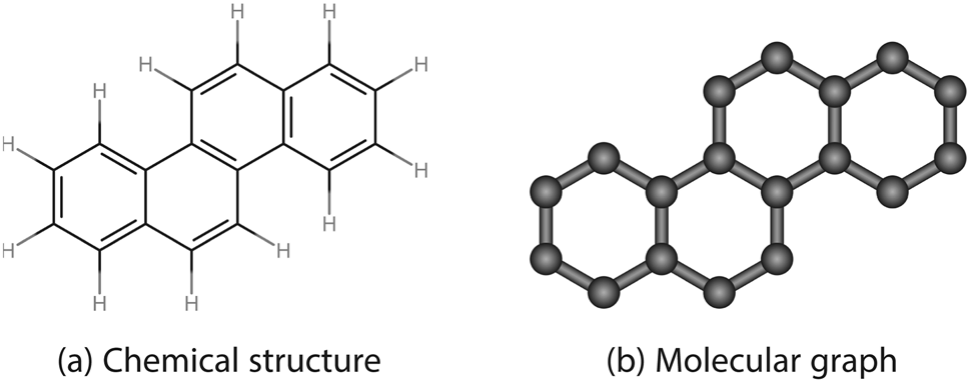
Chemical and molecular graph of cyclic structured drugs.

In general, structures are categorized into three classes/type: polycyclic, acyclic and cyclic. Figures 1, 2, and 3 shows the chemical structure and molecular graph of polycyclic, acyclic and cyclic structured drugs, respectively.

The study of the drugs of several ailments is of great importance in chemical graph theory and this branch of science uses graphs to model a compound and retrieve a lot of data about the compound. In cheminformatics, topological indices are mathematical tools that are used to predict the physicochemical characteristics of drugs or compounds. Many researchers proposed the relationship between physicochemical properties of drugs and topological indices such as entropy indices^15^, M-polynomial indices^16,17^, irregularity indices^18^, ve degree indices^19,20^ and eigenvalue indices^21^.

The classification of topological indices consists of three types: degree-based indices, distance-based indices, and spectrum-based indices. In 1947, Harold Wiener made a significant contribution to the field of chemistry by introducing the concept of a topological index during his research on the boiling point of paraffin^22^. The index in question was commonly known as the path number. The path number was subsequently referred to as the Wiener index, marking the initiation of the investigation into topological indices. The Wiener index is widely recognized as the initial and extensively studied topological index. The class of degree-based topological indices has been extensively studied, particularly in comparison to other classes^23^. The topological properties of metal-organic structures were calculated by Nadeem et al.^24^. Qin et al.^25^ discuss the application of topological indices application for pulmonary cancer drugs. Nasreen et al.^26^ applied topological indices for computing entropy measures of moelcaur netwroks. Ahmad et al.^27,28^ talk about the theoretical analysis of anthracene and phenylene energy. The valency-based topological description for Hexagon Star Networks was established by Koam et al.^29^. Several polynomials, including the Tutte, matching, Schultz, Hosoya, and Zhang-Zhang polynomial, have been proposed. This study focuses on the M-polynomial, demonstrating its role in calculating degree-based indices, analogous to the Hosoya polynomial’s function for distance-based indices.

Introduced by Munir et al. in 2016^30^, the M-polynomial has emerged as a fundamental tool for deriving degree-based invariants. Let $M(G;x,y)=p(x,y)$, where

$$\begin{aligned} & D_x=x\frac{\partial p(x,y)}{\partial x}, \quad D_y=y\frac{\partial p(x,y)}{\partial y}, \quad I_x=\int \limits _{0}^x \frac{p(t,y)}{t}dt, \\ & I_y=\int \limits _{0}^y \frac{p(x,t)}{t}dt, \quad J(p(x,y))=p(x,x), \quad Q_{\alpha }(p(x,y))=x^{\alpha }p(x,y). \end{aligned}$$

Table 1 shows the mathematical form of M-polynomial indices.

**Table 1:** Formulas of M-polynomial indices.

Index name	Notation	Derivation
First Zagreb	${M_1}$	$(D_x+D_y)\cdot (\mathrm {M(G)})|_{x,y=1}$
Second Zagreb	${M_2}$	$(D_xD_y)\cdot (\mathrm {M(G)})|_{x,y=1}$
Augmented Zagreb	*AZI*	$I_x^{3}Q_{-2}JD_x^{3}D_y^3(\mathrm {M(G)})|_{x=1}$
Modified second Zagreb	${^mM_2}$	$(I_xI_y)\cdot (\mathrm {M(G)})|_{x,y=1}$
Harmonic	*H*	$2I_xJ(\mathrm {M(G)})|_{x=1}$
General Randić	${R_{\alpha }}$	$D_x^{\alpha }D_y^{\alpha }(\mathrm {M(G)})|_{x,y=1}$
Inverse-sum	*I*	$I_xJD_xD_y(\mathrm {M(G)})|_{x=1}$
Forgotten	*F*	$(D_x^2+D_y^2)\cdot (\mathrm {M(G)})|_{x,y=1}$
Symmetric division	*SDD*	$(D_xI_y+I_xD_y)\cdot (\mathrm {M(G)})|_{x,y=1}$
Redefined third Zagreb	${ReZG_3}$	$D_x\cdot D_y(D_x+D_y)\cdot \mathrm {M(G)}|_{x,y=1}$

## Methodology

In this section, we outline the methodology employed in this study to compute M-polynomial indices of dextran and chitosan, and their statistical analysis with ADME properties of polycyclic structured drugs. The methodology is divided into two main phases: M-polynomial indices computation and statistical analysis.

### Computation of M-polynomial indices

In Sections “Dextran” and “Chitosan”, we compute the M-polynomial indices for dextran and chitosan using the following steps:

1. The chemical structure of dextran and chitosan is transformed into molecular graph, where atoms serve as vertices and chemical bonds as edges.
2. The vertices and edges of the molecular graphs of dextran and chitosan are partitioned based on the degree.
3. A two variable polynomial proposed to calculate the M-polynomial indices, and graphical representations of the results are plotted using MATLAB software version (R2021a).

### Statistical analysis of M-polynomial indices

To investigate the predictive capability of M-polynomial indices for the ADME-related physico-chemical properties of polycyclic-structured drugs, we performed a comprehensive statistical analysis using state-of-the-art computational techniques. The methodology includes the following key steps:

- A dataset of drugs with polycyclic structures was selected based on structural diversity and relevance to biocompatible polymers such as dextran and chitosan.
- Each drug’s chemical structure was represented as a molecular graph, where atoms correspond to vertices and chemical bonds to edges.
- The molecular graph’s adjacency matrix was generated using the newGraph software (version 1.4).
- A Python-based computational algorithm was developed to calculate M-polynomial indices directly from the adjacency matrix, improving efficiency and eliminating manual errors.
- ADME-related physico-chemical properties (e.g., molecular weight, exact mass, molar volume, surface tension, boiling point) were extracted from public chemical databases such as PubChem and ChemSpider.
- Advanced regression techniques—including Multiple Linear Regression (MLR), Ridge, Lasso, ElasticNet, and Support Vector Regression (SVR)—were employed to model the relationship between M-polynomial indices (independent variables) and the selected physico-chemical properties (dependent variables).
- The predictive performance of each model was evaluated using both cross-validation (to assess internal consistency) and external validation (to test model generalizability), using key metrics such as the Pearson correlation coefficient (*R*), coefficient of determination ($R^2$), root mean squared error (RMSE), and *p*-values.
- Only statistically significant models with robust performance on both validation schemes were retained for final interpretation and biological relevance assessment.

This methodology provides a systematic approach to evaluating M-polynomial indices and their potential significance in understanding the ADME properties of drug compounds.

## Main results

In this chapter, we determined the degree-based M-polynomial indices for dextran and chitosan.

### Dextran

Dextran is a complex, branched carbohydrate composed of glucose molecules linked together in an intricate network. It is produced from the bacterium Leuconostoc mesenteroides and consists of glucose chains that differ in length, forming a heterogeneous structure^31^. Dextran’s molecular weight can vary from 10,000 to 2,000,000 Da (dalton), depending on the type and source. It’s branched structure contributes to its high viscosity and solubility^32^. It is highly hydrophilic, making it an excellent solubilizer and stabilizer^33^.

Dextran is used as a filler, binder, and solubilizer in tablets, capsules, and injectable formulations. Dextran 40 and Dextran 70 are used as plasma volume expanders to treat shock, trauma, and burns^34^. It is used in biotechnology applications, such as protein purification, cell culture, and gene delivery. It is also used as a thickening agent, stabilizer, and texture modifier in food products, such as sauces, dressings, and beverages. It is used as a carrier for targeted drug delivery, allowing for controlled release and improved efficacy^35^. Dextran is used as a scaffold material for tissue engineering, providing a biocompatible and biodegradableframework for cell growth. It works as a contrast agent in diagnostic imaging, such as MRI and CT scans. It is used in wound healing applications^36^, such as dressings and hydrogels, to promote tissue repair and regeneration. Native dextran derived from the bacterium Leuconostoc mesenteroides^37^. Modified dextran chemically modified to improve its properties, such as solubility or biodegradability. Dextran sulfate is a sulfated derivative of dextran, used in biomedical applications^38^.

**Fig. 4:**
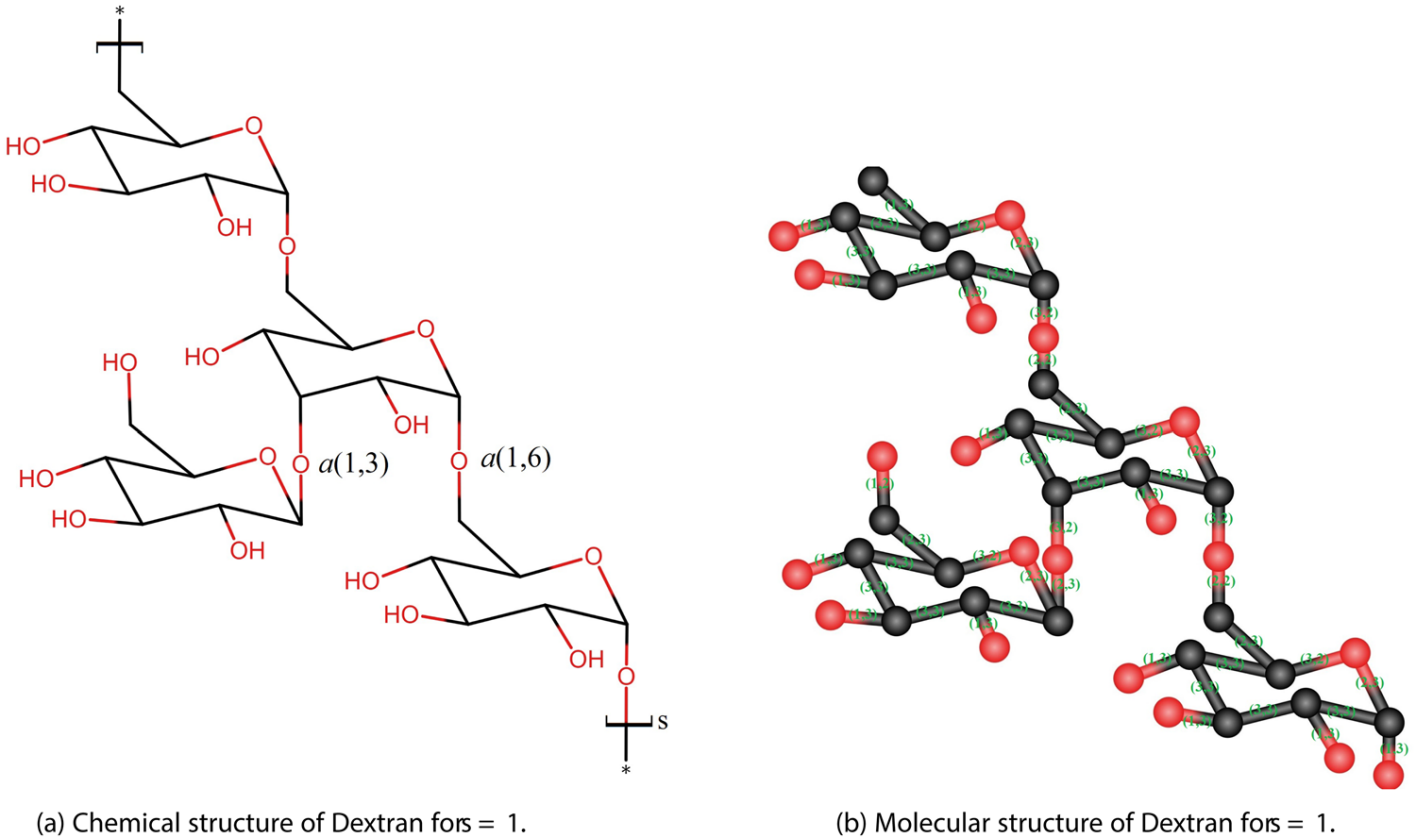
The unit structures of the Dextran.

Dextran is generally considered safe and non-toxic. However, high molecular weight dextrans may cause anaphylactic reactions in some individuals. Figure 4 shows the chemical structure and molecular graph of Dextran for $s=1$, where each single or double bond is represented as an edge. Table 2 shows the partition of edges of dextran.

**Table 2:** Edges partition of dextran.

Notation of an edge	$(d_u,d_v)$	Total number of edges
$\mathscr {E}_{1}$	${(1,\ 3)}$	$11s+2$
$\mathscr {E}_{2}$	${(3,\ 3)}$	16*s*
$\mathscr {E}_{3}$	${(2,\ 3)}$	$17s-2$
$\mathscr {E}_{4}$	${(2,\ 2)}$	$3s-1$
$\mathscr {E}_{5}$	${(1,\ 2)}$	*s*

#### **Theorem 1**

*Let S be a molecular graph of Dextran. Then the M-polynomial of* *S is*

$$\begin{aligned} M(S;x,y)= & \left( 11xy^3+16x^3y^{3}+17x^2y^3+3x^2y^2+xy^2\right) n+2xy^{3}-2x^{2}y^{3}-x^{2}y^{2}. \end{aligned}$$

#### Proof

Suppose *S* be a molecular graph that contain $|V(S)|= 44s$ vertices and $|E(S)|= 48s-1$ edges. *S* have $12s+2$ vertices of degree 1,$12s-2$ vertices of degree 2 and 20*s* vertices of degree 3. Let us consider the edge set $N_{(i,j)}=\{ {w_i}{w_j}\in E(G) | {\Lambda _{w_i}}=i, \quad {\Lambda _{w_j}}=j$} in *S* such that the degree of vertex $w_i$ is *i* and the degree of vertex $w_j$ is *j*. The edges divided into five parts based on degrees of the end vertices, i.e.,

$$N_{(1,3)}=\{ w_1w_3\in E | {\Lambda _{w_1}}=1, {\Lambda _{w_3}}=3\}$$, $$N_{(3,3)}=\{ w_3w_3\in E | {\Lambda _{w_3}}=3, {\Lambda _{w_3}}=3\}$$, $$N_{(2,3)}$$ $$=\{w_2w_3\in E$$ $${\Lambda _{w_2}}=2,$$ $${\Delta _{w_3}}=3\}$$,    $$N_{(2,2)}=$$ $$\{w_2w_2\in E | {\Lambda _{w_2}}=2,$$ $${\Lambda _{w_2}}=2\}$$, $$N_{(1,2)}$$ $$\{w_1w_2\in E | {\Lambda _{w_1}}=1,$$ $${\Lambda _{w_2}}=2\}$$.    

Then we have

$$|N_{(1,3)}|=11s+2$$, $$|N_{(3,3)}|=16s$$, $$|N_{(2,3)}|=17s-2$$, $$|N_{(2,2)}|=3s-1$$, $$|N_{(1,2)}|=s$$.

By the definition of M-polynomial, we conclude

$$\begin{aligned} M(S;x,y)= & \sum \limits _{i\le j}|N_{(i,j)}|x^iy^j,\\ M(S;x,y)= & |N_{(1,3)}|x\cdot y^3+|N_{(3,3)}|x^3\cdot y^3+|N_{(2,3)}|x^2\cdot y^3+|N_{(2,2)}|x^2\cdot y^2+|N_{(1,2)}|x\cdot y^2. \end{aligned}$$

By using the measures of the absolute value of the set $N_{(i,j)}$, we get

$$\begin{aligned} M(S;x,y)= & (11s+2)xy^3+(16s)x^3y^3+(17s-2)x^2y^3+(3s-1)x^2y^2+(s)xy^2\\ M(S;x,y)= & \left( 11xy^{3}+16x^{3}y^{3}+17x^{2}y^{3}+3x^{2}y^{2}+xy^{2} \right) s+2xy^{3}-2x^{2}y^{3}-x^{2}y^{2}\\ \end{aligned}$$

#### **Theorem 2**

*Let S be a graph of Dextran. Then*

1. *First Zagreb index* $({M}_1)=240s-6,$
2. *Second Zagreb index* $({M}_2)=293s-10,$
3. *Redefine third Zagreb index* $({ReZG}_3)=1560s-52,$
4. *Forgotten index* $$({F}) =648s-14,$$
5. *General Randić index* $({R}_{\alpha }) =$  $\left( {11}^{\alpha +1}{33}^{\alpha } \right.$ $+(16){48}^{2\alpha }$ $+(17){34}^{\alpha }{51}^{\alpha }$ $+(3){6}^{2\alpha }$ $\left. +{2}^{\alpha } \right) s$ $+{2}^{\alpha +1}{6}^{\alpha }$ $-{2}^{2\alpha +1}{6}^{\alpha }$ $-{2}^{2\alpha },$
6. *Modified second Zagreb index* $(^mM_2)=\frac{343}{36}s+\frac{1}{12},$
7. *Symmetric division index* $({SDI})=\frac{871}{2}s+\frac{1}{3},$
8. *Harmonic index* $$(H) =\frac{297}{15}s-\frac{3}{10},$$
9. *Inverse sum index* $({I}) =\frac{3379}{60}s-\frac{19}{10}.$

#### Proof

From Theorem 1, the degree based M-polynomial for *S* is

$$\begin{aligned} M(S;x,y)= & \left( 11{x}^{1}{y}^{3}+16{x}^{3}{y}^{3}+17{x}^{2}{y}^{3}+3{x}^{2}{y}^{2}+{x}^{1}{y}^{2} \right) s+2{x}^{1}{y}^{3}-2{x}^{2}{y}^{3}-{x}^{2}{y}^{2}. \end{aligned}$$

By utilizing this polynomial, we get

1. The $M_1$ index is
  $$\begin{aligned} (D_x+D_y)M(S;x,y)= & \left( 44x{y}^{3}+96{x}^{3}{y}^{3}+85{x}^{2}{y}^{3}+12{x}^{2} {y}^{2}+3x{y}^{2} \right) s+8x{y}^{3}-10{x}^{2}{y}^{3}-4{x}^{2}{y}^{2}\\ {M}_1= & (D_x+D_y)M(S;x,y)|_{x,\ y=1}\\ {M}_1= & \, 240s-6. \end{aligned}$$
2. The $M_2$ index is
  $$\begin{aligned} D_xD_y(M(S;x,y))= & \left( 33x{y}^{3}+144{x}^{3}{y}^{3}+102{x}^{2}{y}^{3}+12{x}^{2}{y}^{2}+2x {y}^{2} \right) s+6x{y}^{3}-12{x}^{2}{y}^{3}-4{x}^{2}{y}^{2}\\ {M}_2= & \, (D_xD_y)Q(S;x,y)|_{x,\ y=1}\\ {M}_2= &\,293s-10. \end{aligned}$$
3. The $ReZG_3$ index is
  $$\begin{aligned} D_xD_y(D_x+D_y)M(S;x,y)= & \left( 132x{y}^{3}+864{x}^{3}{y}^{3}+510{x}^{2}{y}^{3}+84{x}^{2}{y}^{2}+6x{y}^{2} \right) s+24x{y}^{3}-60{x}^{2}{y}^{3}-16{x}^{2}{y}^{2}\\ {ReZG}_3= & D_xD_y(D_x+D_y)M(S;x,y)|_{x,\ y=1}\\ {ReZG}_3= & 1560s-52. \end{aligned}$$
4. The *F* index is
  $$\begin{aligned} (D_x^2+D_y^2)M(S;x,y)= & \, \left( 110x{y}^{3}+288{x}^{3}{y}^{3}+221{x}^{2}{y}^{3}+24{x}^{2}{y}^{2}+5x{y}^{2} \right) s+20x{y}^{3}-26{x}^{2}{y}^{3}-8{x}^{2}{y}^{2}\\ {F}= & \, (D_x^2+D_y^2)M(S;x,y)_{x,\ y=1}\\ {F}= & \, 648s-14. \end{aligned}$$
5. The $R_{\alpha }$ index is
  $$\begin{aligned} D_x^{\alpha }D_y^{\alpha }(M(S;x,y))& = (11({11}^{\alpha }{33}^{\alpha })x{y}^{3}+16({48}^{\alpha }{48}^{\alpha }){x}^{3}{y}^{3}+17({34}^{\alpha }{51}^{\alpha }){x}^{2}{y}^{3}+3({6}^{\alpha }{6}^{\alpha }){x}^{2}{y}^{2}+({1}^{\alpha }{2}^{\alpha })x{y}^{2})s +2({2}^{\alpha }{6}^{\alpha })x{y}^{3}\\ & \quad - 2({4}^{\alpha }{6}^{\alpha }){x}^{2}{y}^{3}-({2}^{\alpha }{2}^{\alpha }){x}^{2}{y}^{2}\\ {R_{\alpha }} & = D_x^{\alpha }D_y^{\alpha }(M(S;x,y))|_{x,\ y=1}\\ {R_{\alpha }} & = \left( {11}^{\alpha +1}{33}^{\alpha }+16({48}^{2\alpha })+17({34}^{\alpha }{51}^{\alpha })+3({6}^{2\alpha })+({2}^{\alpha }) \right) s+({2}^{\alpha +1}{6}^{\alpha })-({2}^{2\alpha +1}{6}^{\alpha })-({2}^{2\alpha }). \end{aligned}$$
6. The $^m{{M}_2}$ index is
  $$\begin{aligned} I_xI_y(M(S;x,y))= & \left( \frac{11}{3}x{y}^{3}+\frac{16}{9}{x}^{3}{y}^{3}+\frac{17}{6}{x}^{2}{y}^{3}+\frac{3}{4}{x}^{2} {y}^{2}+\frac{1}{2}x{y}^{2} \right) s+\frac{2}{3}x{y}^{3}-\frac{1}{3}{x}^{2}{y}^{3}-\frac{1}{4}{x}^{2}{y}^{2}\\ ^m{{M}_2}= & I_xI_y(M(S;x,y))|_{x,\ y=1}\\ ^m{{M}_2}= & \frac{343}{36}s+\frac{1}{12}. \end{aligned}$$
7. The *SDI* index is
  $$\begin{aligned} (D_xI_y+I_xD_y)M(S;x,y)= & \left( \frac{1100}{3}x{y}^{3}+32{x}^{3}{y}^{3}+\frac{85}{3}{x}^{2}{y}^{3}+6x^{2}{y}^{2} +\frac{5}{2}x{y}^{2} \right) s+\frac{20}{3}x{y}^{3}-\frac{13}{3}{x}^{2}{y}^{3}-2{x}^{2}{y}^{2}\\ {SDI}= & (D_xI_y+I_xD_y)M(S;x,y)|_{x,\ y=1}\\ {SDI}= & \frac{871}{2}s+\frac{1}{3}. \end{aligned}$$
8. The *H* index is
  $$\begin{aligned} 2I_xJ(M(S;x,y))= & \left( \frac{16}{3}{x}^{6}+\frac{34}{5}{x}^{5}+7{x}^{4}+\frac{2}{3}{x}^{3} \right) s+\frac{1}{2}{x}^{4} -\frac{4}{5}x^{5}\\ H= & 2I_xJ(M(S;x,y))|_{x=1}\\ H= & \frac{297}{15}s-\frac{3}{10}. \end{aligned}$$
9. The *I* index is
  $$\begin{aligned} I_xJD_xD_y(M(S;x,y))= & \left( \frac{144}{6}{x}^{6}+\frac{102}{5}{x}^{5}+\frac{45}{4}{x}^{4}+\frac{2}{3}{x}^{3} \right) s+\frac{2}{4}{x}^{4}-\frac{12}{5}{x}^{5}\\ {I}= & I_xJD_xD_y(M(S;x,y))|_{x=1}\\ {I}= & \frac{3379}{60}s-\frac{19}{10}. \end{aligned}$$

**Fig. 5:**
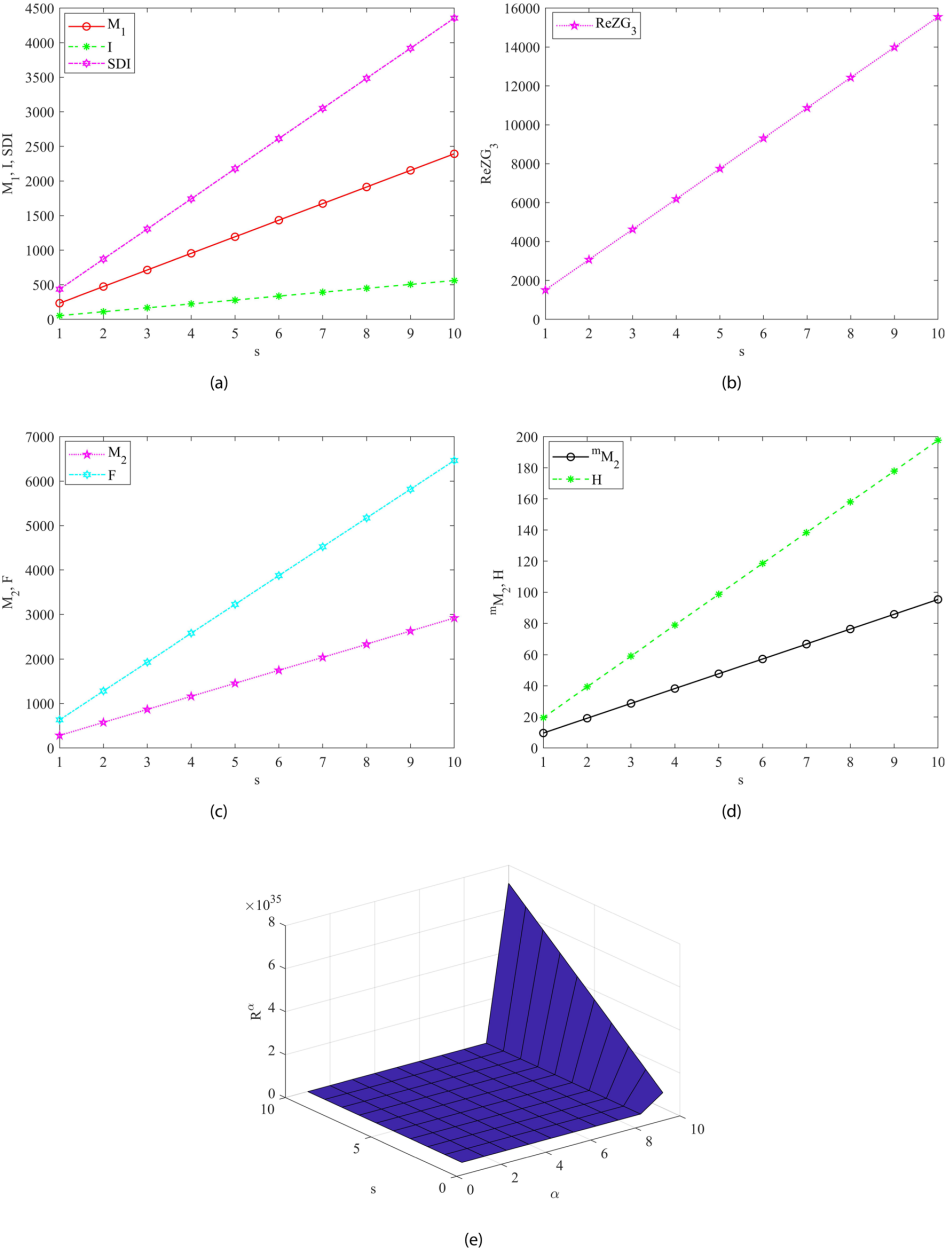
Graphical representation of all M-polynomial indices for Dextran as mentioned in Theorem 2. In (**a**) The first Zagreb, Symmetric division and Inverse sum index, (**b**) The refined third Zagreb and Augmented Zagreb index (**c**) The second Zagreb and Forgotten index (**d**) The Modified second Zagreb and Harmonic index and (**e**) The Randic index for $\alpha \in [1,10]$.

The graphic representation of Theorem 2 is depicted in Fig. 5.

### Chitosan

Chitosan is a biodegradable, non-toxic, and biocompatible polysaccharide derived from the shells of crustaceans, such as shrimp and crabs. It’s a popular ingredient in various industries due to its unique properties. Chitosan has a positive charge, making it suitable for binding to negatively charged molecules. Chitosan is biodegradable^39^, meaning it can be easily broken down by enzymes in the body. It is non-toxic and hypoallergenic, making it suitable for use in medical and food applications. It is soluble in acidic solutions, but insoluble in neutral and alkaline solutions.

Chitosan is used in drug delivery systems, wound dressings, and as a carrier for vaccines. It is used as a food additive, thickening agent, and stabilizer in products such as juices, beverages, and dairy products. Chitosan is used in skincare products, hair care products, and as a thickening agent in cosmetics. It works as a flocculant to remove impurities and contaminants from water. It is used in tissue engineering, wound healing, and as a scaffold for cell growth^40,41^.

Chitosan has antimicrobial properties, making it effective against bacteria, fungi, and viruses. It has immunomodulatory effects, meaning it can stimulate or suppress the immune system as needed. Low molecular weight chitosan has a lower molecular weight and is more soluble in water. High molecular weight chitosan has a higher molecular weight and is more effective as a thickening agent. Oligochitosan is a shorter-chain version of chitosan and has improved solubility and bioavailability.

**Fig. 6:**
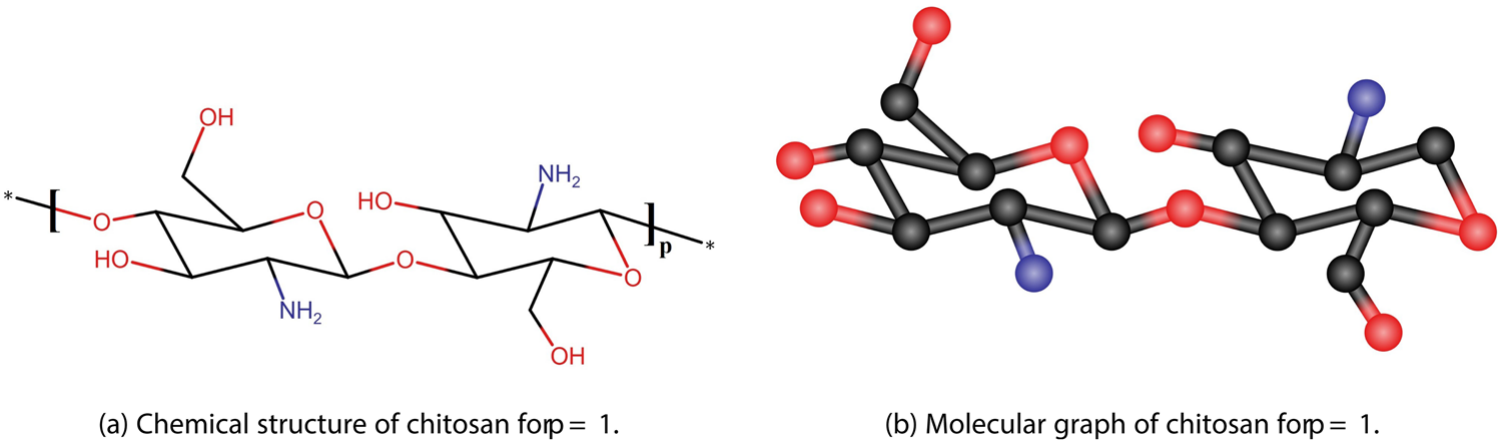
The unit structures of the chitosan.

**Fig. 7:**
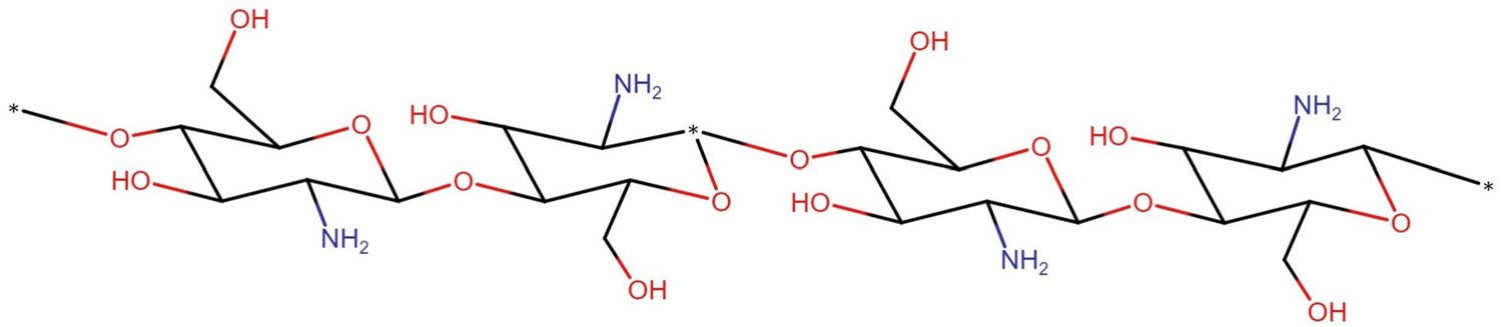
Chemical structure of the chitosan for $p=2$.

Let us consider *S* the molecular structure of chitosan. Figure 6 shows the chemical structure and the molecular graph of chitosan for $p=1$, where each single or double bond is represented as an edge. Figure 7 present the chemical structure of chitosan for $p=2$. Table 3 shows the partition of edges of chitosan.

**Table 3:** Edges partition of chitosan.

Notation of an edge	$(d_u,d_v)$	Total number of edges
$\mathscr {E}_{1}$	${(1,\ 2)}$	2*p*
$\mathscr {E}_{2}$	${(1,\ 3)}$	$4p+1$
$\mathscr {E}_{2}$	${(2,\ 2)}$	1
$\mathscr {E}_{3}$	${(2,\ 3)}$	$10p-2$
$\mathscr {E}_{4}$	${(3,\ 3)}$	$8p-1$

#### **Theorem 3**

*Let P be a molecular graph of chitosan. Then the M-polynomial of* *P is*

$$\begin{aligned} M'(P;x,y)= & \left( 2x{y}^{2}+4x{y}^{3}+10{x}^{2}{y}^{3}+8{x}^{3}{y}^{3}\right) p+x{y}^{3}-2{x}^{2}{y}^{3}-x^3y^3+x^2y^2. \end{aligned}$$

#### Proof

   Let *P* be a graph containing $|V(P)|=22p+1$ vertices and $|E(P)|=24p-1$ edges. The edges are partitioned as follows:

$$|N_{(1,2)}|=2p$$, $$|N_{(1,3)}|=4p+1$$, $$|N_{(2,2)}|=1$$, $$|N_{(2,3)}|=10p-2$$, $$|N_{(3,3)}|=8p-1$$.

By definition, we can deduce

$$\begin{aligned} M' (P;x,y)= & \sum \limits _{i\le j}|N_{(i,j)}|x^iy^j\\= & |N_{(1,2)}|x^1\cdot y^2+|N_{(1,3)}|x^1\cdot y^3+|N_{(2,2)}|x^2\cdot y^2+|N_{(2,3)}|x^2\cdot y^3+|N_{(3,3)}|x^3\cdot y^3. \end{aligned}$$

We acquired

$$\begin{aligned} M' (P;x,y)= & \left( 2x{y}^{2}+4x{y}^{3}+10{x}^{2}{y}^{3}+8{x}^{3}{y}^{3}\right) p+x{y}^{3}-2{x}^{2}{y}^{3}-x^3y^3+x^2y^2. \end{aligned}$$

#### **Theorem 4**

*Let P be a molecular graph of chitosan. Then*

1. *First Zagreb index* $({M_1})=120p - 8,$
2. *Second Zagreb index* $({M_2})=148p - 14,$
3. *Forgotten index* $$({F}) =324p - 26,$$
4. *Redefined third Zagreb index* $({ReZG_3})=792p - 86,$
5. *General Randić index* $({R_{\alpha }})=\left( 2p(12x^2 + 15x + 8) - 3x^2 - 4x + 3\right) ^\alpha \left( 2y^2(p(24y + 1) - 3y + 1)\right) ^\alpha ,$
6. *Modified second Zagreb index* $({^mM_2})=\dfrac{44}{9}p + \dfrac{5}{36},$
7. *Symmetric division index* $({SDI})=56p - 1,$
8. *Harmonic index* $$({H}) =10p - \dfrac{2}{15},$$
9. *Inverse sum index* $({I}) =\dfrac{85}{3}p - \dfrac{43}{20}$.

#### Proof

   From Theorem 3, the degree-based M-polynomial for *P* is

$$\begin{aligned} p(x,y)= & \left( 2x{y}^{2}+4x{y}^{3}+10{x}^{2}{y}^{3}+8{x}^{3}{y}^{3}\right) p+x{y}^{3}-2{x}^{2}{y}^{3}-x^3y^3+x^2y^2. \end{aligned}$$

Using this polynomial, we get

**Fig. 8:**
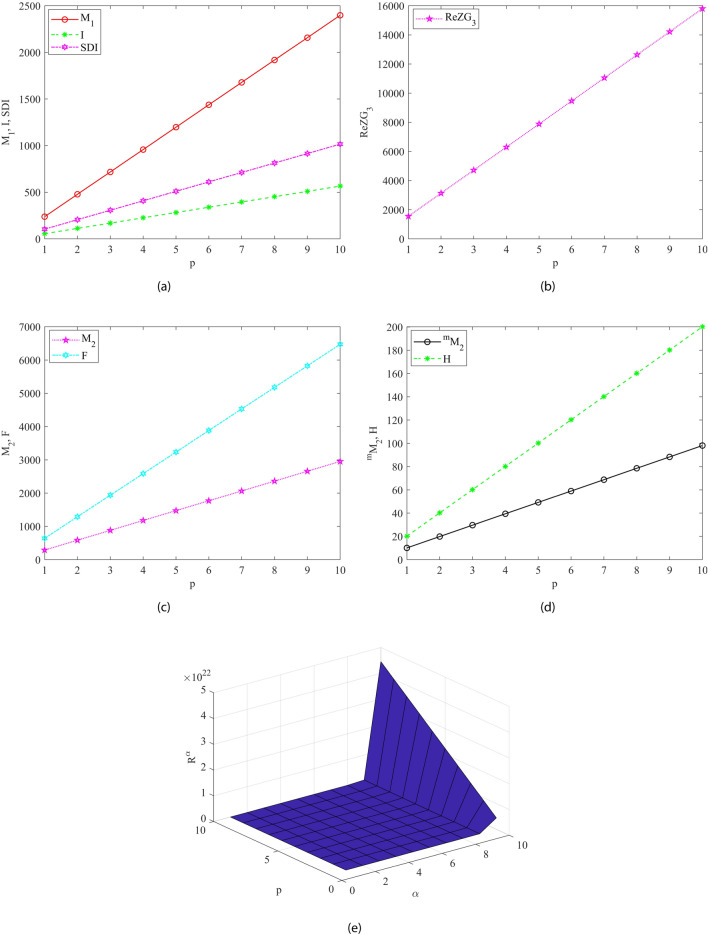
Graphical visualization of all M-polynomial indices for chitosan as mentioned in Theorem 4. In (**a**) The first Zagreb, Symmetric division and Inverse sum index, (**b**) The refined third Zagreb and Augmented Zagreb index, (**c**) The second Zagreb and Forgotten index, (**d**) The Modified second Zagreb and Harmonic index and (**e**) The Randic index for $\alpha \in [1,10]$.

1. The $M_1$ index is
  $$\begin{aligned} (D_x + D_y)p(x, y)= & x \left( p(24x^2y^3 + 20xy^3 + 4y^3 + 2y^2) - 3x^2y^3 - 4xy^3 + 2xy^2 + y^3 \right) \\ & +\, y \left( p(24x^3y^2 + 30x^2y^2 + 12xy^2 + 4xy) - 3x^3y^2 - 6x^2y^2 + 2x^2y + 3xy^2\right) \\ M_1= & (D_x + D_y)p(x,y)|_{x=1,y=1} = 120p - 8 \end{aligned}$$
2. The $M_2$ index is
  $$\begin{aligned} D_xD_y(p(x,y))= & xy \left( p(72x^2y^2 + 60xy^2 + 12y^2 + 4y) - 9x^2y^2 - 12xy^2 + 4xy + 3y^2 \right) \\ M_2= & D_xD_y(p(x,y))|_{x=1,y=1} = 148p - 14 \end{aligned}$$
3. The *F* index is
  $$\begin{aligned} (D_x^2 + D_y^2)p(x, y)= & x \left( p(24x^2y^3 + 20xy^3 + 4y^3 + 2y^2) - 3x^2y^3 - 4xy^3 + 2xy^2 + y^3 \right) \\ & + \, x^2 \left( p(48xy^3 + 20y^3) - 6xy^3 - 4y^3 + 2y^2 \right) \\ & + \, y \left( p(24x^3y^2 + 30x^2y^2 + 12xy^2 + 4xy) - 3x^3y^2 - 6x^2y^2 + 2x^2y + 3xy^2 \right) \\ & + \, y^2 \left( p(48x^3y + 60x^2y + 24xy + 4x) - 6x^3y - 12x^2y + 2x^2 + 6xy \right) \\ F= & (D_x^2 + D_y^2)p(x,y)|_{x=1,y=1} = 324p - 26 \end{aligned}$$
4. The $ReZG_3$ index is
  $$\begin{aligned} D_xD_y(D_x + D_y)p(x,y)= & xy \left( 2p(72x^2y^2 + 60xy^2 + 12y^2 + 4y) - 18x^2y^2 - 24xy^2 + 8xy \right) \\ & + \, x^2 \left( p(144xy^2 + 60y^2) - 18xy^2 - 12y^2 + 4y \right) \\ & + \, y^2 \left( p(144x^2y + 120xy + 24y + 4) - 18x^2y - 24xy + 4x + 6y \right) \\ ReZG_3= & D_xD_y(D_x + D_y)p(x,y)|_{x=1,y=1} = 792p - 86 \end{aligned}$$
5. The $R_{\alpha }$ index is
  $$\begin{aligned} R_{\alpha }= & \left( x\left( 2p(12x^2 + 15x + 8) - 3x^2 - 4x + 3\right) \right) ^\alpha \cdot \left( 2y^2(p(24y + 1) - 3y + 1)\right) ^\alpha \end{aligned}$$
6. The $^mM_2$ index is
  $$\begin{aligned} I_xI_y(p(x,y))= & \, y^3\left( \frac{8p x^3}{9} + \frac{5p x^2}{3} + \frac{4p x}{3} - \frac{x^3}{9} - \frac{x^2}{3} + \frac{x}{3}\right) + y^2\left( px + \frac{x^2}{4}\right) \\ ^mM_2= & \, I_xI_y(p(x,y))|_{x=1,y=1} = \frac{44}{9}p + \frac{5}{36} \end{aligned}$$
7. The *SDI* index is
  $$\begin{aligned} (D_xI_y + I_xD_y)p(x,y)= & \, x^3(8py^3 - y^3) + x^2(15py^3 - 3y^3 + y^2) \\ & + \, x\left( y^3(8px^2 + \frac{20p x}{3} + \frac{4p}{3} - x^2 - \frac{4x}{3} + \frac{1}{3}) + y^2(p + x)\right) \\ & + \, x(12py^3 + 4py^2 + 3y^3) \\ SDI= & (D_xI_y + I_xD_y)p(x,y)|_{x=1,y=1} = 56p - 1 \end{aligned}$$
8. The *H* index is
  $$\begin{aligned} 2I_xJ(p(x))= & \frac{4p x^3}{3} + 2x^6\left( \frac{4p}{3} - \frac{1}{6} \right) + 2x^5\left( 2p - \frac{2}{5} \right) + 2x^4\left( p + \frac{1}{2} \right) \\ H= & 2I_xJ(p(x))|_{x=1} = 10p - \frac{2}{15} \end{aligned}$$
9. The *I* index is
  $$\begin{aligned} J(D_xD_y(p(x,y)))= & \, p(4x^3 + 12x^4 + 60x^5 + 72x^6) + 7x^4 - 12x^5 - 9x^6 \\ \frac{J(D_xD_y(p(x,y)))}{x}= & \, p(4x^2 + 12x^3 + 60x^4 + 72x^5) + 7x^3 - 12x^4 - 9x^5 \\ I_xJ(D_xD_y(p(x)))= & \int _0^1 \left[ p(4x^2 + 12x^3 + 60x^4 + 72x^5) + 7x^3 - 12x^4 - 9x^5 \right] \frac{1}{x} dx \\= & \int _0^1 \left( p(4x^2 + 12x^3 + 60x^4 + 72x^5) + 7x^3 - 12x^4 - 9x^5 \right) \frac{1}{x} dx \\= & \frac{85}{3}p - \frac{43}{20} \\ I= & I_xJ(D_xD_y(p(x)))|_{x=1} = \frac{85}{3}p - \frac{43}{20} \end{aligned}$$
  The graphic representation of Theorem 4 is depicted in Fig. 8.

### Python code for the computation of M-polynomial indices

The computation of M-polynomial indices values is a complex and time-consuming task that involves several error-prone steps. Traditionally, this process begins with converting the chemical structure of a molecule into a molecular graph, where atoms are represented as vertices and chemical bonds as edges. Next, degrees are assigned to each vertex, and edges are partitioned based on the degrees of their end vertices. The frequency of edges is then used to generate a polynomial in two variables, usually x and y. Following partial derivative w.r.t. x and y, this polynomial is then integrated w.r.t. x and y. The M-polynomial indices are then determined using the resultant polynomial, necessitating further mathematical operations.

In addition to being time-consuming, this manual procedure is prone to human mistake, especially when working with big and intricate molecular structures. We suggest a novel Python method that effectively computes M-polynomial indices by utilizing the molecular graph’s adjacency matrix in order to address these issues. Our method eliminates human mistake, drastically cuts down computation time from days to minutes, and gives researchers a dependable and quick result by automating the calculating process.

**Listing 1:**
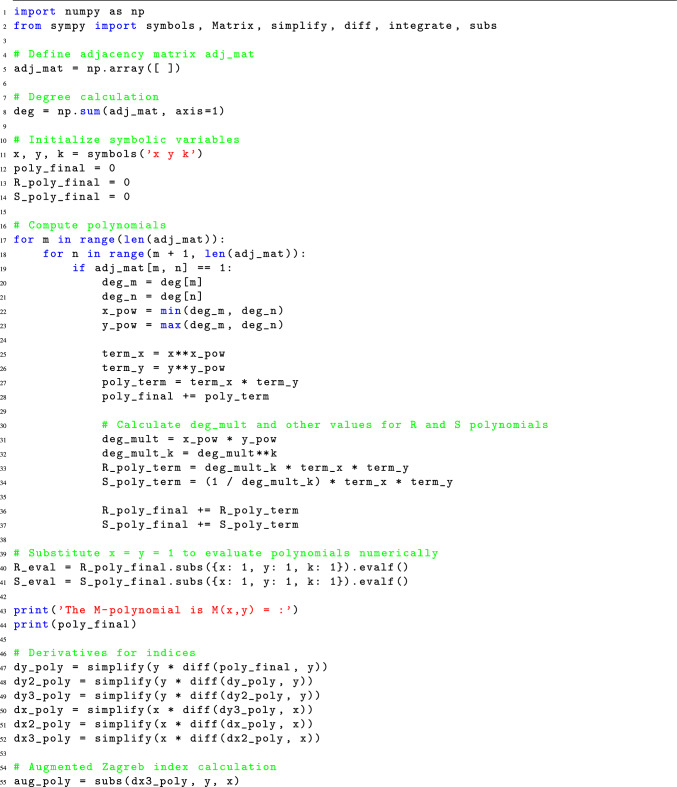

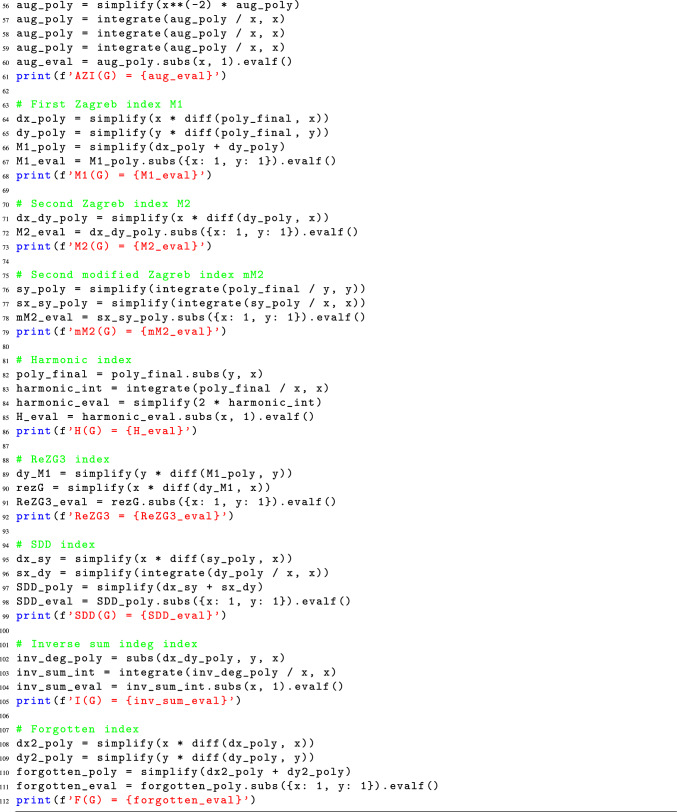
Python code for M-Polynomial indices calculation from graphs.

## Statistical analysis of M-polynomial indices

      Statistical analysis an essential method for predicting the ADME properties of molecules using M-polynomial indices. These M-polynomial indices, obtained from molecular graphs, encapsulate structural information that corresponds with the physical qualities of pharmaceuticals. The statistical analysis between the ADME properties of polycyclic structured therapeutics and their M-polynomial indices is currently under development in this domain. To calculate the M-polynomial indices, we initially transform the chemical structure of the drug into a molecular graph. The list of selected drug molecules along with their corresponding PubChem CIDs and ChemSpider IDs are shown in Table 4. The M-polynomial indices values of polycyclic structured drugs are shown in Table 5 and their physical attributes are listed in Table 6.

**Table 4:** List of selected drug molecules along with their corresponding PubChem CIDs and ChemSpider IDs.

Sr.#	Drug name	PubChem CID	ChemSpider ID	Sr. #	Drug name	PubChem CID	ChemSpider ID
1	Acetaminophen	1983	1906	24	Lisinopril	5362119	4514933
2	Amitriptyline	2160	2075	25	Lofexidine	30668	28460
3	Amlodipine	2162	2077	26	Loratadine	3957	3820
4	Amoxicillin	33613	31006	27	Lyrica	5486971	4589156
5	Ativan	3958	3821	28	Melatonin	896	872
6	Atorvastatin	60823	54810	29	Meloxicam	54677470	10442740
7	Azithromycin	447043	10482163	30	Metformin	4091	3949
8	Benzonatate	7699	7413	31	Methadone	4095	3953
9	Brilinta	9871419	8047109	32	Methotrexate	126941	112728
10	Buprenorphine	644073	559124	33	Metoprolol	4171	4027
11	Cephalexin	27447	25541	34	Naltrexone	5360515	4514524
12	Ciprofloxacin	2764	2662	35	Naproxen	156391	137720
13	Citalopram	2771	2669	36	Narcan	5284596	4447644
14	Clindamycin	446598	393915	37	Omeprazole	4594	4433
15	Clonazepam	2802	2700	38	Otezla	11561674	9736448
16	Cyclobenzaprine	2895	21168	39	Pantoprazole	4679	4517
17	Doxycycline	54671203	10482106	40	Prednisone	5865	5656
18	Fentanyl	3345	3228	41	Probuphine	3033050	559124
19	Gabapentin	3446	3328	42	Qulipta	72163100	59718640
20	Hydrochlorothiazide	3639	3513	43	Tramadol	33741	31105
21	Ibuprofen	3672	3544	44	Trazodone	5533	5332
22	Imbruvica	24821094	26637187	45	Xanax	2118	2034
23	Jardiance	11949646	10123957

**Table 5:** The M-polynomial indices for polycyclic drug molecules.

Drugs/Indices	$\textbf{AZI}$	$\mathbf {M_1}$	$\mathbf {M_2}$	$\mathbf {^mM_2}$	$\textbf{H}$	$\mathbf {ReZG_3}$	$\textbf{Sch}$	$\textbf{Gut}$	$\textbf{SDI}$	$\textbf{I}$	$\textbf{F}$
Acetaminophen	74.125	50	53	2.5	4.9	248	106	496	27	11.45	124
Amitriptyline	188.3125	108	126	4.6944	10.0333	624	252	1248	50	26.1	268
Amlodipine	240.625	136	160	6.6389	12.9667	820	320	1640	66	32.4	346
Amoxicillin	209.9397	138	168	5.2639	10.8857	918	336	1836	68.25	31.5476	390
Ativan	182.4531	112	133	4.5556	9.6667	682	266	1364	53	26.5	292
Atorvastatin	345.5156	210	245	9.0833	18.9	1238	490	2476	101.3333	49.7	538
Azithromycin	400.9291	274	327	11.2361	22.1095	1798	654	3596	142.25	61.3405	786
Benzonatate	334.7656	174	180	10.4444	20.5667	770	360	1540	87.3333	42.7833	372
Brilinta	335.4062	196	238	7.9444	16.8667	1236	476	2472	89.3333	46.9333	512
Buprenorphine	379.4833	236	319	6.8194	15.5667	1940	638	3880	103.5	54.2833	738
Cephalexin	210.7656	128	155	5.25	10.9	818	310	1636	61	30.2	340
Ciprofloxacin	224.625	134	164	5.1389	11.1667	860	328	1720	61	32	354
Citalopram	196.5386	122	145	5.0278	10.5714	762	290	1524	58.1667	28.5952	326
Clindamycin	218.75	136	161	6.1389	12	844	322	1688	68	31.5833	362
Clonazepam	190.4531	116	137	4.8056	10.1667	698	274	1396	55	27.5	300
Cyclobenzaprine	206.8772	126	148	5.2361	11.0524	766	296	1532	60.9167	29.5476	334
Doxycycline	295.2174	184	237	6.7778	13.8905	1360	474	2720	87.5	42.5929	532
Fentanyl	221.5469	124	142	5.75	12.0667	686	284	1372	57.6667	30.1167	300
Gabapentin	86.75	56	62	2.8333	5.4	316	124	632	29.3333	12.7	150
Hydrochlorothiazide	129.6561	94	111	3.4306	7.0714	618	222	1236	50.0833	20.5119	282
Ibuprofen	103.6562	70	77	3.3889	6.5667	380	154	760	37.6667	15.95	180
Imbruvica	310.4844	178	213	7.2778	15.8	1078	426	2156	80	43.0667	452
Jardiance	273.8438	164	194	6.8333	14.4667	984	388	1968	76.6667	39.0667	422
Lisinopril	233.8281	138	156	6.7222	13.4333	766	312	1532	68.6667	32.7167	342
Lofexidine	132.2969	80	92	3.5833	7.4	458	184	916	39	18.95	202
Loratadine	251.0938	144	172	6	12.8667	872	344	1744	65.3333	34.7667	366
Lyrica	61.5	44	44	2.6667	4.6667	204	88	408	26.6667	9.6667	110
Melatonin	141.5312	84	96	3.8889	7.9333	472	192	944	40.6667	19.9667	210
Meloxicam	198.8419	126	153	4.8889	10.2381	830	306	1660	61.3333	29.1286	350
Metformin	44.2656	36	36	2.1111	3.6333	174	72	348	23	7.65	94
Methadone	194.3626	114	135	5.3194	10.5905	716	270	1432	55.5833	26.8429	300
Methotrexate	259.9531	168	193	7.2222	14.8667	970	386	1940	83.6667	39.1	436
Metoprolol	138.125	84	89	4.5	8.7667	408	178	816	44	19.7167	200
Naltrexone	292.3325	176	237	5.0764	12.0262	1396	474	2792	73.5833	41.5238	526
Naproxen	140.2969	86	100	3.8333	7.7667	506	200	1012	42	20.2167	222
Narcan	276.3325	164	221	5.0764	11.4929	1312	442	2624	69.5833	38.5905	492
Omeprazole	211.0781	126	150	5.3889	11.1	770	300	1540	59.3333	29.8833	328
Otezla	267.1267	168	199	7.0417	14.2667	1048	398	2096	83.4167	38.7667	458
Pantoprazole	223.6875	134	157	5.8611	12.0667	792	314	1584	63.6667	31.7833	344
Prednisone	235.7242	146	185	5.2778	11.2095	1048	370	2096	67.8333	33.8405	422
Probuphine	379.4833	236	319	6.8194	15.5667	1940	638	3880	103.5	54.2833	738
Qulipta	383.681	246	302	8.9306	19.2381	1640	604	3280	116.5833	56.9786	690
Tramadol	156.7684	96	112	4.2917	8.6714	586	224	1172	47.4167	22.3286	256
Trazodone	236.3125	138	162	5.6944	12.4333	804	324	1608	63	33.3	346
Xanax	211.0938	122	148	4.75	10.5	760	296	1520	54.3333	29.5	314

**Table 6:** The ADME properties of polycyclic structure drugs.

Drugs/Properties	MW	EM	PSA	C	BP	EoV	FP	IoR	MR	P	ST	MV
Acetaminophen	151.16	151.0633	49.3	139	387.8	66.2	188.4	1.619	42.4	16.8	52.8	120.9
Amitriptyline	277.4	277.183	3.2	331	398.2	64.9	174	1.628	91.5	36.3	47	257.8
Amlodipine	408.9	408.1452	99.9	647	527.2	80.2	272.6	1.546	105.4	41.8	44.4	333
Amoxicillin	365.4	365.1045	158	590	743.2	113.7	403.3	1.702	91.5	36.3	85.3	236.2
Ativan	321.2	320.0119	61.7	443	543.6	86.5	282.6	1.694	81	32.1	56	211
Atorvastatin	558.6	558.253	112	822	722.2	110.7	390.6	1.603	155.2	61.5	46	451.9
Azithromycin	749	748.5085	180	1150	822.1	136	451	1.537	197.6	78.3	50.6	632.7
Benzonatate	603.7	603.3619	121	565	649	95.7	346.3	1.495	160.6	63.7	39.8	550.7
Brilinta	522.6	522.1861	164	736	777.6	118.7	424	1.744	126.3	50.1	63.3	311.9
Buprenorphine	467.6	467.3036	62.2	869	667.4	116.5	401	1.632	131.4	52.1	58.2	368.3
Cephalexin	347.4	347.094	138	600	727.4	111.5	393.7	1.7	89.4	35.4	78.5	231.3
Ciprofloxacin	331.34	331.1332	72.9	571	581.8	91.5	305.6	1.655	83.3	33	67.4	226.8
Citalopram	324.4	324.1638	36.3	466	428.3	68.3	212.8	1.591	92.1	36.5	49.9	272.6
Clindamycin	425	424.1799	128	502	628.1	106.5	333.6	1.574	107.9	42.8	56.2	327.2
Clonazepam	315.71	315.0411	87.3	491	524.5	79.8	271	1.701	81.2	32.2	61.8	209.9
Cyclobenzaprine	275.4	275.1674	3.2	365	405.9	65.8	177.8	1.646	91.1	36.1	48.5	251.1
Doxycycline	444.4	444.1533	182	956	762.6	116.5	415	1.737	109	43.2	99.2	271.1
Fentanyl	336.5	336.2202	23.6	391	466.2	72.8	185.8	1.585	103.7	41.1	45.7	309.4
Gabapentin	171.24	171.1259	63.3	162	314.4	61.1	144	1.489	46.7	18.5	47.1	161.8
Hydrochlorothiazide	297.7	296.9645	135	494	577	86.4	302.7	1.632	62.7	24.9	62	175.8
Ibuprofen	206.28	206.1307	37.3	203	319.6	59.3	216.7	1.519	60.8	24.1	38.1	200.3
Imbruvica	440.5	440.1961	99.2	678	715	104.5	386.2	1.696	126.1	50	56.5	327.5
Jardiance	450.9	450.1445	109	558	664.5	102.7	355.7	1.628	114.4	45.4	59.4	322.4
Lisinopril	405.5	405.2264	133	550	666.4	102.9	356.9	1.578	107.5	42.6	60.4	323.9
Lofexidine	259.13	258.0327	33.6	263	421.5	64.9	208.7	1.611	64.7	25.6	43.4	186.3
Loratadine	382.9	382.1448	42.4	569	531.3	80.7	275.1	1.614	105.9	42	53.3	303.5
Lyrica	159.23	159.1259	63.3	123	274	56.4	119.5	1.465	44.1	17.5	37.9	159.6
Melatonin	232.28	232.1212	54.1	270	512.8	78.4	264	1.6	67.6	26.8	46.7	197.6
Meloxicam	351.4	351.0347	136	628	692	103.1	392.2	1.72	86	34.1	85.3	217.7
Metformin	129.16	129.1014	91.5	132	172.5	40.9	58.1	1.576	33.4	13.2	50.8	100.8
Methadone	309.4	309.2093	20.3	346	423.7	67.8	126.5	1.538	95.9	38	37.1	306.5
Methotrexate	454.4	454.1713	211	704	533.2	101.1	389.7	1.738	119	47.2	96.5	295.7
Metoprolol	267.36	267.1834	50.7	215	398.6	68.5	194.9	1.508	77.1	30.6	37.1	258.7
Naltrexone	341.4	341.1627	70	621	558.1	88.4	291.4	1.709	90.1	35.7	77.1	230.9
Naproxen	230.26	230.0943	46.5	277	403.9	69.1	154.5	1.609	66.5	26.4	47.5	192.3
Narcan	327.4	327.1471	70	594	532.8	85.1	276.1	1.691	87.3	34.6	72.1	228.1
Omeprazole	345.4	345.1147	96.3	453	600	89.3	316.7	1.669	94	37.3	75.2	251.9
Otezla	460.5	460.1304	127	825	741.3	108.1	402.1	1.612	115.9	45.9	60.6	333.3
Pantoprazole	383.4	383.0751	106	490	586.9	87.6	308.7	1.643	91.4	36.2	73.5	252.7
Prednisone	358.4	358.178	91.7	764	573.7	98.7	314.8	1.604	94.1	37.3	58.6	273.6
Probuphine	467.6	467.3036	62.2	869	583.7	99.2	323.4	1.632	131.4	52.1	58.2	368.3
Qulipta	603.5	603.1705	104	1110	693.9	101.7	373.5	1.624	138	54.7	63.6	390.9
Tramadol	263.37	263.1885	32.7	282	388.1	67.2	188.5	1.533	78	30.9	39.7	251.4
Trazodone	371.9	371.1513	42.4	611	528.5	80.3	273.4	1.671	104.2	41.3	55.1	278.8
Xanax	308.8	308.0829	43.1	434	509	77.9	261.6	1.711	88.2	35	52.2	225.6

To investigate the relationship between M-polynomial indices and the ADME-relevant physico-chemical properties of polycyclic drug compounds, we employed a range of regression techniques including Multiple Linear Regression (MLR), Ridge Regression (RR), Lasso Regression (LR), ElasticNet Regression (ENR), and Support Vector Regression (SVR). The predictive performance of each model was evaluated using cross-validation and external test metrics across several endpoints, as presented in Tables 7, 8, 9, 10, 11, 12, 13, 14, 15, 16, 17, and 18. Key evaluation metrics included the coefficient of determination ($R^2$), Pearson correlation coefficient (R), root mean square error (RMSE), and the associated *p*-value, which collectively assess model accuracy, robustness, and generalizability.

**Table 7:** Cross validation and external test metrics for molecular weight prediction.

Cross_Validation_Metrics					
Model	R_cv	${R}^{2}$_corr_cv	${R}^{2}$_cv	RMSE_cv	*p*-value_cv
LinearRegression	0.9797	0.9598	0.9595	25.2638	0.0000
Ridge	0.9789	0.9582	0.9580	25.7354	0.0000
Lasso	0.9798	0.9600	0.9599	25.1298	0.0000
ElasticNet	0.9656	0.9324	0.8768	44.0675	0.0000
SVR	0.4361	0.1902	0.0279	123.7966	0.0078

External_Test_Metrics					
Model	R_external	${R}^{2}$_corr_external	${R}^{2}$_external	RMSE_external	*p*-value_external
LinearRegression	0.9928	0.9856	0.9802	16.8165	0.0000
Ridge	0.9917	0.9835	0.9768	18.2030	0.0000
Lasso	0.9929	0.9858	0.9794	17.1658	0.0000
ElasticNet	0.9813	0.9630	0.8949	38.7643	0.0000
SVR	0.8061	0.6499	− 0.0115	120.2297	0.0087

**Table 8:** Cross validation and external test metrics for exact mass prediction.

Cross_Validation_Metrics					
Model	R_cv	${R}^{2}$_corr_cv	${R}^{2}$_cv	RMSE_cv	*p*-value_cv
LinearRegression	0.9799	0.9602	0.9600	25.0984	0.0000
Ridge	0.9791	0.9587	0.9585	25.5755	0.0000
Lasso	0.9800	0.9605	0.9604	24.9652	0.0000
ElasticNet	0.9659	0.9329	0.8772	43.9716	0.0000
SVR	0.4386	0.1923	0.0282	123.7200	0.0075

External_Test_Metrics					
Model	R_external	${R}^{2}$_corr_external	${R}^{2}$_external	RMSE_external	*p*-value_external
LinearRegression	0.9930	0.9860	0.9807	16.6193	0.0000
Ridge	0.9919	0.9839	0.9773	18.0184	0.0000
Lasso	0.9931	0.9862	0.9798	16.9745	0.0000
ElasticNet	0.9816	0.9636	0.8953	38.6725	0.0000
SVR	0.8066	0.6506	− 0.0105	120.1478	0.0086

**Table 9:** Cross validation and external test metrics for complexity prediction.

Cross_Validation_Metrics					
Model	R_cv	${R}^{2}$_corr_cv	${R}^{2}$_cv	RMSE_cv	*p*-value_cv
LinearRegression	0.9158	0.8386	0.8350	98.3589	0.0000
Ridge	0.9179	0.8425	0.8416	96.3627	0.0000
Lasso	0.9158	0.8387	0.8356	98.1694	0.0000
ElasticNet	0.9229	0.8518	0.8047	106.9996	0.0000
SVR	− 0.0675	0.0046	− 0.0243	245.0642	0.6956

External_Test_Metrics					
Model	R_external	${R}^{2}$_corr_external	${R}^{2}$_external	RMSE_external	*p*-value_external
LinearRegression	0.9501	0.9027	0.8641	93.3095	0.0001
Ridge	0.9504	0.9033	0.8599	94.7387	0.0001
Lasso	0.9501	0.9027	0.8631	93.6751	0.0001
ElasticNet	0.9517	0.9058	0.7666	122.3074	0.0001
SVR	0.7868	0.6190	− 0.1309	269.2017	0.0119

**Table 10:** Cross validation and external test metrics for boiling point prediction.

Cross_Validation_Metrics					
Model	R_cv	${R}^{2}$_corr_cv	${R}^{2}$_cv	RMSE_cv	*p*-value_cv
LinearRegression	0.7175	0.5149	0.4993	105.5155	0.0000
Ridge	0.7208	0.5195	0.5102	104.3663	0.0000
Lasso	0.7174	0.5146	0.5010	105.3343	0.0000
ElasticNet	0.7297	0.5324	0.5183	103.4971	0.0000
SVR	− 0.1141	0.0130	− 0.0630	153.7415	0.5075

External_Test_Metrics					
Model	R_external	${R}^{2}$_corr_external	${R}^{2}$_external	RMSE_external	*p*-value_external
LinearRegression	0.8985	0.8073	0.7661	65.4260	0.0010
Ridge	0.8982	0.8067	0.7635	65.7845	0.0010
Lasso	0.8985	0.8074	0.7653	65.5421	0.0010
ElasticNet	0.8959	0.8027	0.7036	73.6468	0.0011
SVR	0.8519	0.7257	0.0064	134.8536	0.0036

**Table 11:** Cross validation and external test metrics for enthalpy of vaporization prediction.

Cross_Validation_Metrics					
Model	R_cv	${R}^{2}$_corr_cv	${R}^{2}$_cv	RMSE_cv	*p*-value_cv
LinearRegression	0.7732	0.5978	0.5836	13.4512	0.0000
Ridge	0.7776	0.6046	0.5969	13.2343	0.0000
Lasso	0.7712	0.5948	0.5909	13.3335	0.0000
ElasticNet	0.7900	0.6241	0.5968	13.2360	0.0000
SVR	0.5214	0.2719	0.1514	19.2033	0.0011

External_Test_Metrics					
Model	R_external	${R}^{2}$_corr_external	${R}^{2}$_external	RMSE_external	*p*-value_external
LinearRegression	0.8169	0.6674	0.5844	11.2230	0.0072
Ridge	0.8170	0.6675	0.5900	11.1468	0.0072
Lasso	0.8168	0.6672	0.6017	10.9867	0.0072
ElasticNet	0.8175	0.6683	0.6127	10.8344	0.0071
SVR	0.8294	0.6879	0.2866	14.7033	0.0057

**Table 12:** Cross validation and external test metrics for flash point prediction.

Cross_Validation_Metrics					
Model	R_cv	${R}^{2}$_corr_cv	${R}^{2}$_cv	RMSE_cv	*p*-value_cv
LinearRegression	0.6915	0.4782	0.4593	72.1795	0.0000
Ridge	0.6948	0.4827	0.4708	71.4129	0.0000
Lasso	0.6910	0.4775	0.4619	72.0115	0.0000
ElasticNet	0.7026	0.4936	0.4831	70.5733	0.0000
SVR	0.1275	0.0162	− 0.0098	98.6460	0.4588

External_Test_Metrics					
Model	R_external	${R}^{2}$_corr_external	${R}^{2}$_external	RMSE_external	*p*-value_external
LinearRegression	0.8895	0.7911	0.7509	41.0717	0.0013
Ridge	0.8894	0.7910	0.7507	41.0894	0.0013
Lasso	0.8895	0.7912	0.7510	41.0640	0.0013
ElasticNet	0.8888	0.7900	0.7113	44.2130	0.0014
SVR	0.8602	0.7399	− 0.0066	82.5606	0.0029

**Table 13:** Cross validation and external test metrics for molar refractivity prediction.

Cross_Validation_Metrics					
Model	R_cv	${R}^{2}$_corr_cv	${R}^{2}$_cv	RMSE_cv	*p*-value_cv
LinearRegression	0.9875	0.9751	0.9750	5.1966	0.0000
Ridge	0.9873	0.9748	0.9743	5.2673	0.0000
Lasso	0.9875	0.9751	0.9743	5.2709	0.0000
ElasticNet	0.9761	0.9529	0.8865	11.0732	0.0000
SVR	0.7356	0.5411	0.1237	30.7652	0.0000

External_Test_Metrics					
Model	R_external	${R}^{2}$_corr_external	${R}^{2}$_external	RMSE_external	*p*-value_external
LinearRegression	0.9755	0.9515	0.9093	7.6804	0.0000
Ridge	0.9742	0.9490	0.9126	7.5364	0.0000
Lasso	0.9761	0.9527	0.9252	6.9745	0.0000
ElasticNet	0.9625	0.9263	0.9123	7.5533	0.0000
SVR	0.8927	0.7968	0.2192	22.5308	0.0012

**Table 14:** Cross validation and external test metrics for polarization prediction.

Cross_Validation_Metrics					
Model	R_cv	${R}^{2}$_corr_cv	${R}^{2}$_cv	RMSE_cv	*p*-value_cv
LinearRegression	0.9874	0.9750	0.9749	2.0624	0.0000
Ridge	0.9873	0.9747	0.9742	2.0910	0.0000
Lasso	0.9870	0.9742	0.9674	2.3528	0.0000
ElasticNet	0.9767	0.9539	0.8761	4.5863	0.0000
SVR	0.8016	0.6426	0.3150	10.7839	0.0000

External_Test_Metrics					
Model	R_external	${R}^{2}$_corr_external	${R}^{2}$_external	RMSE_external	*p*-value_external
LinearRegression	0.9755	0.9517	0.9095	3.0415	0.0000
Ridge	0.9742	0.9491	0.9128	2.9848	0.0000
Lasso	0.9770	0.9546	0.9431	2.4114	0.0000
ElasticNet	0.9629	0.9272	0.9094	3.0426	0.0000
SVR	0.8953	0.8016	0.5337	6.9028	0.0011

**Table 15:** Cross validation and external test metrics for surface tension prediction.

Cross_Validation_Metrics					
Model	R_cv	${R}^{2}$_corr_cv	${R}^{2}$_cv	RMSE_cv	*p*-value_cv
LinearRegression	0.2678	0.0717	0.0360	15.9451	0.1143
Ridge	0.2625	0.0689	0.0450	15.8703	0.1220
Lasso	0.2127	0.0452	0.0336	15.9647	0.2130
ElasticNet	0.1785	0.0319	0.0305	15.9905	0.2975
SVR	0.3834	0.1470	0.0342	15.9596	0.0210

External_Test_Metrics					
Model	R_external	${R}^{2}$_corr_external	${R}^{2}$_external	RMSE_external	*p*-value_external
LinearRegression	0.9202	0.8468	0.5902	5.8289	0.0004
Ridge	0.9220	0.8502	0.5830	5.8797	0.0004
Lasso	0.9038	0.8168	0.5377	6.1909	0.0004
ElasticNet	0.8862	0.7853	0.4311	6.8682	0.0015
SVR	0.7506	0.5634	0.2658	7.8022	0.0198

**Table 16:** Cross validation and external test metrics for molar volume prediction.

Cross_Validation_Metrics					
Model	R_cv	${R}^{2}$_corr_cv	${R}^{2}$_cv	RMSE_cv	*p*-value_cv
LinearRegression	0.9474	0.8975	0.8974	33.5698	0.0000
Ridge	0.9464	0.8956	0.8942	34.0841	0.0000
Lasso	0.9480	0.8987	0.8986	33.3699	0.0000
ElasticNet	0.9155	0.8381	0.7788	49.2976	0.0000
SVR	0.3832	0.1468	− 0.0147	105.5774	0.0211

External_Test_Metrics					
Model	R_external	${R}^{2}$_corr_external	${R}^{2}$_external	RMSE_external	*p*-value_external
LinearRegression	0.9450	0.8930	0.6403	37.4086	0.0001
Ridge	0.9442	0.8916	0.6591	36.4206	0.0001
Lasso	0.9444	0.8920	0.6405	37.3997	0.0001
ElasticNet	0.9305	0.8658	0.7921	28.4414	0.0003
SVR	0.8045	0.6473	0.0294	61.4505	0.0089

**Table 17:** Cross validation and external test metrics for polar surface area prediction.

Cross_Validation_Metrics					
Model	R_cv	${R}^{2}$_corr_cv	${R}^{2}$_cv	RMSE_cv	*p*-value_cv
LinearRegression	0.3912	0.1530	0.1235	47.8991	0.0183
Ridge	0.3914	0.1532	0.1310	47.6937	0.0183
Lasso	0.4006	0.1605	0.1444	47.3243	0.0155
ElasticNet	0.3746	0.1403	0.1402	47.4413	0.0244
SVR	0.0057	0.0000	− 0.0356	52.0664	0.9737

External_Test_Metrics					
Model	R_external	${R}^{2}$_corr_external	${R}^{2}$_external	RMSE_external	*p*-value_external
LinearRegression	0.6012	0.3614	0.2120	33.7409	0.0868
Ridge	0.6017	0.3621	0.2095	33.7951	0.0865
Lasso	0.6009	0.3611	0.2139	33.7006	0.0870
ElasticNet	0.6004	0.3605	0.1761	34.5021	0.0874
SVR	0.5491	0.3015	− 0.1137	40.1131	0.1258

**Table 18:** Cross validation and external test metrics for index of refraction prediction.

Cross_Validation_Metrics					
Model	R_cv	${R}^{2}$_corr_cv	${R}^{2}$_cv	RMSE_cv	*p*-value_cv
LinearRegression	0.3136	0.0983	0.0550	0.0677	0.0626
Ridge	0.3062	0.0938	0.0687	0.0673	0.0693
Lasso	− 0.3356	0.1126	− 0.0673	0.0720	0.0454
ElasticNet	− 0.3356	0.1126	− 0.0673	0.0720	0.0454
SVR	0.1588	0.0252	0.0196	0.0690	0.3549

External_Test_Metrics					
Model	R_external	${R}^{2}$_corr_external	${R}^{2}$_external	RMSE_external	*p*-value_external
LinearRegression	0.3700	0.1369	0.1269	0.0711	0.3270
Ridge	0.3828	0.1465	0.1292	0.0710	0.3092
Lasso	NaN	NaN	− 0.0308	0.0773	NaN
ElasticNet	NaN	NaN	− 0.0308	0.0773	NaN
SVR	0.6933	0.4807	0.1891	0.0685	0.0384

By applying various regression techniques, we developed predictive models for several physicochemical properties, including molar volume (MV), boiling point (BP), molar refractivity (MR), flash point (FP), surface tension (ST), enthalpy of vaporization (EoV), polarization (P), exact mass (EM), molecular weight (MW), and molecular complexity (C). Below, we present only the most significant regression equations corresponding to each property for clarity and conciseness.

$$\begin{aligned} \text {Lasso:} \quad&\text {MW} = 356.4858 + (106.2236)\cdot ^mM_2(G) + (21.8570)\cdot ReZG_3 \\ \text {Lasso:} \quad&\text {EM} = 356.1307 + (106.1814)\cdot ^mM_2(G) + (21.8621)\cdot ReZG_3 \\ \text {Ridge:} \quad&\text {C} = 512.3056 + (85.0235)\cdot ^mM_2(G) + (154.8383)\cdot ReZG_3 \\ \text {Lasso:} \quad&\text {C} = 512.3056 + (83.1872)\cdot ^mM_2(G) + (159.4224)\cdot ReZG_3 \\ \text {Lasso:} \quad&\text {BP} = 549.8833 + (72.4610)\cdot ^mM_2(G) + (51.8536)\cdot ReZG_3 \\ \text {ElasticNet:} \quad&\text {EoV} = 87.6861 + (6.8399)\cdot ^mM_2(G) + (7.2291)\cdot ReZG_3 \\ \text {Linear:} \quad&\text {FP} = 287.5806 + (42.0713)\cdot ^mM_2(G) + (38.9649)\cdot ReZG_3 \\ \text {Lasso:} \quad&\text {FP} = 287.5806 + (41.4850)\cdot ^mM_2(G) + (38.3746)\cdot ReZG_3 \\ \text {Ridge:} \quad&\text {MR} = 96.0722 + (27.0874)\cdot ^mM_2(G) + (6.3619)\cdot ReZG_3 \\ \text {Lasso:} \quad&\text {MR} = 96.0722 + (27.7290)\cdot ^mM_2(G) + (5.0903)\cdot ReZG_3 \\ \text {Lasso:} \quad&\text {P} = 38.0861 + (10.6400)\cdot ^mM_2(G) + (1.6598)\cdot ReZG_3 \\ \text {Linear:} \quad&\text {ST} = 57.9389 + (-4.1947)\cdot ^mM_2(G) + (8.4483)\cdot ReZG_3 \\ \text {Ridge:} \quad&\text {ST} = 57.9389 + (-3.6952)\cdot ^mM_2(G) + (7.8804)\cdot ReZG_3 \\ \text {ElasticNet:} \quad&\text {MV} = 276.6583 + (58.0554)\cdot ^mM_2(G) + (18.4464)\cdot ReZG_3 \end{aligned}$$

## Discussion of results

In this study, both cross-validation and external validation metrics were computed to rigorously evaluate the predictive performance of regression models. The cross-validation metrics include the Pearson correlation coefficient ($R_{\text {cv}}$), the coefficient of determination from correlation ($R^2_{\text {corr\_cv}}$), standard coefficient of determination ($R^2_{\text {cv}}$), root mean square error ($\hbox {RMSE}_{\text {cv}}$), and the associated *p*-value (P-$\hbox {value}_{\text {cv}}$). These metrics were used to assess the internal consistency and robustness of the models across resampling folds. In parallel, external test metrics−namely $R_{\text {external}}$, $R^2_{\text {corr\_external}}$, $R^2_{\text {external}}$, $\hbox {RMSE}_{\text {external}}$, and P-$\hbox {value}_{\text {external}}$—were calculated using unseen data to evaluate the generalizability and predictive strength of each model outside the training dataset. These comprehensive evaluations ensure that the reported models are not only statistically significant but also practically reliable for modeling the physico-chemical properties of polycyclic drug molecules using M-polynomial indices.

- Table 7 presents the performance of five machine learning models developed to predict the molecular weight of biocompatible polysaccharides using M-polynomial indices as descriptors. The cross-validation results show that Lasso Regression performs best with $R^2_{\text {cv}} = 0.9599$ and RMSE = 25.13, closely followed by Linear and Ridge Regression. All three linear models demonstrate strong predictive ability, indicating a linear relationship between the M-polynomial descriptors and molecular weight.
  ElasticNet shows relatively weaker performance ($R^2_{\text {cv}} = 0.8768$), likely due to its mixed regularization approach. The SVR model performs poorly in both cross-validation and external testing, suggesting it is unsuitable for this dataset. On external test data, Lasso again performs best ($R^2_{\text {external}} = 0.9794$, RMSE = 17.16), reinforcing its robustness and generalization capacity.
  The extremely low *p*-values across all linear models confirm statistical significance. These results suggest that M-polynomial-based topological indices are powerful predictors of molecular weight. Overall, Lasso Regression is the most suitable model due to its high accuracy and built-in feature selection capability, supporting the effectiveness of topological descriptors in QSPR modeling.
- Table 8 summarizes the performance metrics for predicting the exact mass of biocompatible polysaccharides using different regression models based on M-polynomial indices. Among the models evaluated, Lasso Regression again yields the highest performance during cross-validation ($R^2_{\text {cv}} = 0.9604$, RMSE = 24.97), suggesting that it effectively captures the linear relationship between molecular descriptors and exact mass while avoiding overfitting through regularization. Linear and Ridge Regression also perform comparably well with $R^2_{\text {cv}} > 0.958$, confirming the reliability of linear models for this task.
  ElasticNet displays moderate performance, while the SVR model performs poorly across all metrics, including a significantly higher RMSE (123.72) and a very low $R^2_{\text {cv}}$ of 0.0282. External test metrics reinforce the findings from cross-validation: Lasso Regression achieves the best test accuracy ($R^2_{\text {external}} = 0.9798$, RMSE = 16.97), followed closely by Linear Regression ($R^2_{\text {external}} = 0.9807$).
  All linear models yield highly significant *p*-values ($p < 0.0001$), indicating that the observed correlations are statistically robust. The SVR model again fails to generalize, as reflected by a negative $R^2_{\text {external}}$, suggesting overfitting or model mismatch. Overall, the results validate the predictive strength of M-polynomial indices and highlight Lasso Regression as a robust and interpretable model for predicting exact mass.
- Table 9 presents the results of cross-validation and external testing for predicting the Complexity of biocompatible polysaccharides using M-polynomial indices as independent variables. The linear models (Linear Regression, Ridge, and Lasso) performed consistently well in both validation and testing phases. Ridge Regression slightly outperformed the others during cross-validation with $R^2_{\text {cv}} = 0.8416$ and a relatively low RMSE of 96.36. Lasso and Linear Regression followed closely, with $R^2_{\text {cv}}$ values above 0.83 and RMSEs below 99.
  Interestingly, ElasticNet showed high correlation ($R^2_{\text {corr\_cv}} = 0.8518$) but a comparatively lower predictive strength ($R^2_{\text {cv}} = 0.8047$), suggesting possible overfitting or instability in capturing Complexity. Support Vector Regression (SVR), in contrast, failed to model the data effectively, with negative $R^2_{\text {cv}}$ and extremely high RMSE (245.06), indicating poor generalization.
  In external test performance, Ridge and Lasso remained robust with $R^2_{\text {external}} \approx 0.86$ and RMSEs below 95, confirming model generalizability. ElasticNet showed reduced test accuracy ($R^2_{\text {external}} = 0.7666$), while SVR again underperformed, exhibiting a negative $R^2_{\text {external}}$ and the highest RMSE (269.20). All linear models reported statistically significant *p*-values ($< 0.0001$), affirming the validity of correlations. These results underscore the effectiveness of linear approaches, particularly Ridge and Lasso, for modeling structural Complexity using M-polynomial indices.
- Table 10 provides insight into the predictive capability of M-polynomial indices for estimating the boiling point of biocompatible polysaccharides. The linear models demonstrate moderate performance during cross-validation, with $R^2_{\text {cv}}$ values ranging from 0.499 (Linear Regression) to 0.518 (ElasticNet), and RMSEs between 103 and 106. ElasticNet slightly outperforms others in capturing the variability, suggesting it may better balance the bias-variance trade-off in this context.
  Interestingly, Support Vector Regression (SVR) performs poorly with a negative $R^2_{\text {cv}}$ and a high RMSE of 153.74, indicating underfitting and weak model generalization. In contrast, all linear models yield statistically significant correlations ($p < 0.0001$), affirming the reliability of their predictive patterns.
  External test results further validate these observations. The linear models (especially Lasso and Linear Regression) maintain $R^2_{\text {external}}$ values above 0.76, with Lasso achieving the lowest RMSE (65.54). SVR again underperforms dramatically, with $R^2_{\text {external}} = 0.0064$ and RMSE = 134.85, reaffirming its incompatibility for this property. Overall, the moderate performance of linear models and poor SVR outcomes suggest that boiling point may depend on non-linear or unmodeled structural features beyond M-polynomial descriptors.
- Table 11 summarizes the cross-validation and external testing metrics for predicting the enthalpy of vaporization of biocompatible polysaccharides. Among the linear models, ElasticNet shows the highest cross-validation correlation ($R_{\text {cv}} = 0.7900$) and corrected $R^2_{\text {corr\_cv}} = 0.6241$, along with a relatively low RMSE (13.24), indicating a slight advantage in fitting the training data. Ridge and Lasso also perform comparably well with $R^2_{\text {cv}}$ values above 0.59 and significant *p*-values ($<0.0001$), confirming meaningful associations between M-polynomial indices and enthalpy of vaporization.
  Support Vector Regression (SVR) underperforms in cross-validation with a low $R^2_{\text {cv}} = 0.1514$ and a high RMSE of 19.20, suggesting poor learning capability from the given descriptors. However, in external testing, SVR surprisingly achieves a higher correlation coefficient ($R_{\text {external}} = 0.8294$) than the linear models, though its $R^2_{\text {external}}$ (0.2866) and RMSE (14.70) indicate unstable and inconsistent prediction quality.
  The linear models maintain better consistency between training and testing phases. ElasticNet again leads external performance with the lowest RMSE (10.83) and $R^2_{\text {external}} = 0.6127$. Overall, while all models exhibit statistically significant results ($p < 0.01$), ElasticNet offers the most balanced and generalizable performance, supporting its suitability for enthalpy prediction using topological descriptors.
- Table 12 provides the cross-validation and external test results for predicting flash point using M-polynomial indices as independent variables. During cross-validation, ElasticNet outperformed other models with a correlation coefficient $R_{\text {cv}} = 0.7026$ and the highest $R^2_{\text {cv}} = 0.4831$, while also maintaining the lowest RMSE of 70.57. All linear models (Linear Regression, Ridge, Lasso, ElasticNet) exhibited statistically significant predictive capabilities ($p < 0.0001$), confirming meaningful relationships with the M-polynomial descriptors.
  Support Vector Regression (SVR), however, showed very poor performance in cross-validation, with a near-zero $R^2_{\text {cv}}$ (-0.0098) and a high RMSE (98.65), indicating its inability to capture the underlying pattern in the training data.
  For external testing, all linear models achieved strong correlation coefficients ($R_{\text {external}} \approx 0.889$) and consistent $R^2_{\text {external}}$ values around 0.75, indicating good generalization. Notably, Lasso achieved the best RMSE (41.06), closely followed by Linear Regression and Ridge. Although SVR showed an improvement in $R_{\text {external}} = 0.8602$, its $R^2_{\text {external}}$ was negative, suggesting overfitting or model instability. Overall, linear models−particularly Lasso and ElasticNet−demonstrated more reliable and interpretable performance for flash point prediction using topological indices.
- Table 13 presents the performance of various regression models for predicting molar refractivity based on M-polynomial indices. The linear models, particularly Linear Regression, Ridge, and Lasso, demonstrated excellent performance during cross-validation, with high correlation coefficients ($R_{\text {cv}} > 0.987$), high $R^2_{\text {cv}}$ values (above 0.974), and very low RMSE values (around 5.2). All models exhibited statistically significant *p*-values ($p < 0.0001$), confirming the strong relationship between the selected topological descriptors and molar refractivity.
  Among these, Lasso achieved the lowest RMSE (6.97) on the external test set, along with the highest $R^2_{\text {external}} = 0.9252$, suggesting strong predictive generalizability. Although ElasticNet showed relatively strong cross-validation results ($R^2_{\text {cv}} = 0.8865$), its performance slightly declined during external testing compared to Ridge and Lasso. In contrast, SVR showed weak predictive power, with a much lower $R^2_{\text {cv}} = 0.1237$ and high RMSE both in cross-validation (30.76) and testing (22.53), highlighting its inefficiency for this property.
  Overall, linear models, especially Lasso and Ridge, provide highly reliable and interpretable predictions for molar refractivity based on M-polynomial descriptors, validating the robustness of these topological indices for modeling physico-chemical properties.
- Table 14 presents the cross-validation and external test performance of various regression models for predicting the polarization of biocompatible polysaccharides using M-polynomial indices. All linear models−Linear Regression, Ridge, and Lasso−performed exceptionally well, showing high cross-validation correlation coefficients ($R_{\text {cv}} > 0.987$) and $R^2_{\text {cv}}$ values exceeding 0.967. Their RMSE values were relatively low (ranging from 2.06 to 2.35), and *p*-values remained highly significant ($p < 0.0001$), indicating strong model reliability.
  On the external test set, Lasso regression achieved the best generalization with an $R^2_{\text {external}}$ of 0.9431 and the lowest RMSE of 2.41, signifying robust predictive accuracy. Ridge and Linear Regression also maintained high predictive performance with $R^2_{\text {external}} > 0.90$. ElasticNet, while comparable in training, slightly underperformed in external testing relative to Lasso.
  In contrast, SVR yielded significantly lower predictive performance with $R^2_{\text {external}} = 0.5337$ and a notably higher RMSE of 6.90, reflecting its limited suitability for this task. The consistent success of linear models highlights the strong linear correlation between M-polynomial descriptors and the polarization property, further confirming the validity of these topological indices in capturing essential chemical information.
- Table 15 summarizes the predictive performance of five regression models for surface tension using M-polynomial indices. Unlike other physico-chemical properties, surface tension exhibited relatively weak predictive relationships in cross-validation. All models showed low $R_{\text {cv}}$ values (below 0.39), and their $R^2_{\text {cv}}$ values were close to zero. Moreover, the *p*-values for most models were statistically insignificant ($p > 0.05$), indicating a lack of strong correlation between M-polynomial descriptors and surface tension during training.
  However, a contrasting trend is observed in the external test set. Linear models such as Linear Regression, Ridge, and Lasso achieved high correlation coefficients ($R_{\text {external}} > 0.90$), though the $R^2_{\text {external}}$ values were relatively moderate (ranging from 0.43 to 0.59), suggesting that while they captured some trend, the explained variance remained limited. Notably, the Ridge regression showed slightly better generalization ($R^2_{\text {external}} = 0.583$) than others.
  SVR again showed the weakest performance, with the lowest $R^2_{\text {external}} = 0.2658$ and highest RMSE = 7.80, reflecting poor generalization ability. These observations imply that while M-polynomial indices partially capture surface tension trends, additional or alternative molecular descriptors may be required to significantly improve prediction accuracy for this particular property.
- Table 16 presents the predictive performance of different regression models for molar volume using M-polynomial descriptors. During cross-validation, all linear models (Linear Regression, Ridge, Lasso) showed strong correlations, with $R_{\text {cv}}$ values around 0.947-0.948 and high $R^2_{\text {cv}}$ values ($\sim$0.897-0.899), suggesting good model fit. Lasso achieved the lowest RMSE_cv_ (33.37), indicating its slight edge in capturing relevant patterns from the data. ElasticNet also showed reasonably high correlation but with slightly reduced performance ($R^2_{\text {cv}} = 0.7788$). SVR, however, performed poorly with near-zero or negative $R^2_{\text {cv}}$ and significantly higher RMSE.
  In the external test set, a drop in $R^2_{\text {external}}$ is observed for all models except ElasticNet. Surprisingly, although ElasticNet had weaker performance in cross-validation, it yielded the highest $R^2_{\text {external}} = 0.7921$ and the lowest RMSE = 28.44, indicating better generalizability to unseen data. In contrast, the linear models’ external $R^2$ dropped to $\sim$0.64-0.66, suggesting potential mild overfitting. SVR again lagged behind with low correlation and poor predictive ability.
  These findings imply that M-polynomial descriptors effectively capture molar volume trends, and ElasticNet may offer superior robustness by balancing sparsity and regularization, especially when applied to external datasets.
- Table 17 presents the cross-validation and external test results for polar surface area (PSA) prediction using M-polynomial indices. The performance across all models was noticeably weaker compared to other physicochemical properties. During cross-validation, $R_{\text {cv}}$ values remained low (approximately 0.39-0.40) with corresponding $R^2_{\text {cv}}$ values ranging from 0.12 to 0.14. The Root Mean Square Errors (RMSE) were relatively high ($\sim$47), and *p*-values hovered near the 0.05 threshold, reflecting marginal statistical significance.
  In the external test set, the predictive capability remained limited. Linear Regression, Ridge, and Lasso models yielded moderate $R_{\text {external}}$ values ($\sim$0.60), but low $R^2_{\text {external}}$ values (0.17 to 0.21) and non-significant *p*-values ($>0.08$) indicated limited reliability. The SVR model exhibited the weakest performance with a negative $R^2_{\text {external}}$ and the highest RMSE (40.11), underscoring its poor generalization.
  These weak results may be attributed to the inherent structural nature of PSA, which depends on the three-dimensional spatial arrangement and hydrogen bonding capacity of polar atoms−features not fully captured by M-polynomial indices, which are fundamentally two-dimensional topological descriptors. The absence of geometrical and electronic features in M-polynomial calculations limits their ability to represent surface-dependent properties like PSA. These findings underscore the need to incorporate 3D molecular descriptors or hybrid models to enhance prediction accuracy for such spatially sensitive properties.
- Table 18 shows the model performance for predicting the index of refraction (IOR) using M-polynomial indices. Overall, the predictive capability was relatively weak. During cross-validation, Linear Regression and Ridge models produced low correlation coefficients ($R_{\text {cv}} \approx 0.31$) and very low coefficients of determination ($R^2_{\text {cv}} < 0.07$), along with RMSE values around 0.067. Lasso and ElasticNet resulted in negative $R^2_{\text {cv}}$, indicating that they performed worse than a simple mean-based predictor.
  External validation results further confirmed these trends. Linear Regression and Ridge achieved $R^2_{\text {external}}$ values of only $\sim$0.12, with non-significant *p*-values (above 0.30), suggesting weak and unreliable predictive power. Lasso and ElasticNet failed entirely in external testing, returning NaN values−likely due to regularization penalties overpowering weak signals in the data. Interestingly, the SVR model yielded a slightly better $R^2_{\text {external}}$ of 0.1891 with a statistically significant *p*-value (0.0384), though its overall performance remained suboptimal.
  The limitations in modeling IOR may stem from the fact that this property is primarily influenced by molecular polarizability and electronic structure−attributes that are not effectively described by 2D topological metrics like M-polynomials. Consequently, M-polynomial indices exhibit limited sensitivity to the quantum and spectral characteristics essential for accurate refractive index predictions. Future efforts may benefit from integrating quantum-derived descriptors or machine learning frameworks capable of modeling nonlinear dependencies to better capture such complex physicochemical behaviors.

In summary, the regression results reveal that M-polynomial indices exhibit strong predictive capability for various physico-chemical properties of biocompatible polysaccharides, particularly molecular weight, exact mass, molar refractivity, and polarization. Among the tested models, Lasso and Linear Regression consistently performed well with high $R^2$ values and low RMSE, indicating accurate and stable predictions. The significance of the selected model equations lies in their simplicity and interpretability, enabling practical application for compound screening.

Biologically and pharmacologically, these findings are vital because properties such as molecular weight and molar refractivity play a critical role in determining drug solubility, membrane permeability, absorption, and overall bioavailability−key factors for the efficacy of polysaccharide-based therapeutics. For example, molar refractivity and polarizability correlate with drug-receptor interactions and tissue distribution, while polarization influences intermolecular binding and aggregation. The ability to reliably predict these characteristics using M-polynomial indices offers a computationally efficient approach for pre-screening large compound libraries, reducing experimental workload and accelerating lead optimization in drug design.

However, the study also highlights certain limitations. Properties such as surface tension, polar surface area (PSA), and index of refraction showed limited predictability, as reflected by lower $R^2$ values and non-significant *p*-values. This suggests that M-polynomial indices alone may not fully capture the structural complexity underlying these properties. Incorporating additional molecular descriptors−such as 3D conformational parameters, electronic distributions, or quantum chemical features−could enhance predictive accuracy and model generalization for these challenging endpoints. This insight opens avenues for hybrid modeling frameworks that integrate topological and physicochemical features to achieve a more holistic understanding of drug-like behavior.

### Heatmap analysis

**Fig. 9:**
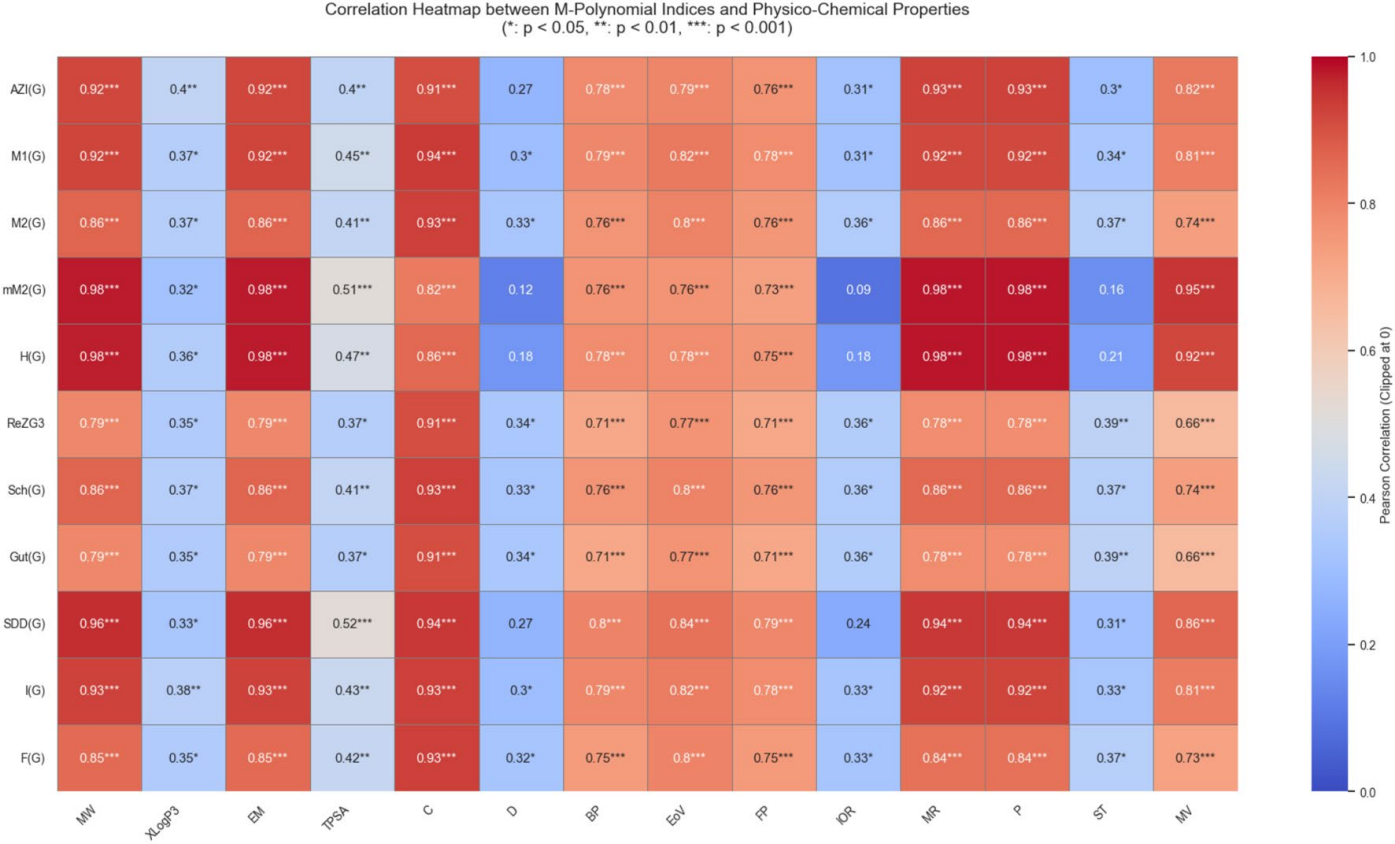
Correlation between the physico-chemical properties and M-polynomial indices only.

To evaluate the linear relationships between M-polynomial indices and selected physico-chemical properties, a Pearson correlation heatmap was constructed, as shown in Fig. 9. In this heatmap, the correlation coefficients are visually encoded using a diverging color scale ranging from blue (correlation = 0) to red (correlation = 1), with all negative correlations clipped to zero for clarity. Annotations within each cell indicate the strength of correlation, with significance levels marked as follows: * for $p < 0.05$, ** for $p < 0.01$, and *** for $p < 0.001$.

The majority of the M-polynomial indices display strong positive correlations (red cells) with several physico-chemical descriptors such as MW, EM, MR, P, EoV, BP, and C, all with high statistical significance ($p < 0.001$). This indicates that the M-polynomial indices are effective in capturing molecular size, shape, and mass-related features.

For instance, indices such as *AZI*, $M_1$,*SDD*, $M_2$, $^mM_2$, and *H* exhibit correlation coefficients above 0.9 with MW, EM, P, C, and MR, all statistically significant at $p < 0.001$, suggesting a robust relationship. Conversely, properties such as TPSA, D, ST, and IoR tend to show weaker correlations, as reflected by lighter blue to white cell shading and fewer significance markers.

Overall, the heatmap confirms that M-polynomial descriptors are highly predictive of key structural and physico-chemical characteristics, particularly those linked to molecular weight and electronic properties.

## Conclusion

This study demonstrates the effective use of M-polynomial indices as topological descriptors for predicting ADME-related physico-chemical properties of polycyclic-structured drugs, with a particular focus on biocompatible polysaccharides such as dextran and chitosan. Various regression models−including Multiple Linear Regression, Ridge, Lasso, ElasticNet, and Support Vector Regression−were employed to develop predictive frameworks. These models were evaluated using both cross-validation metrics and external test metrics, including the coefficient of determination ($R^2$), Pearson correlation coefficient (R), root mean square error (RMSE), and *p*-values.

The results revealed strong and statistically significant correlations between M-polynomial indices and key ADME-related properties such as molecular weight, exact mass, molar refractivity, flash point, surface tension, boiling point, molar volume, molecular complexity, polarization, and enthalpy of vaporization. The consistency between cross-validation and external validation results confirms the robustness and generalizability of the proposed models.

Specifically:

- *Ridge regression* achieved the highest predictive accuracy for molar refractivity, surface tension, and complexity.
- *Multiple Linear Regression* demonstrated strong performance for flash point and surface tension.
- *ElasticNet regression* was most effective in predicting molar volume and enthalpy of vaporization.
- *Lasso regression* provided optimal predictions for molecular weight, exact mass, complexity, flash point, molar refractivity, boiling point, and polarization.

To support wider applications and reproducibility, a Python-based tool was developed for computing M-polynomial indices, enhancing computational efficiency and reducing manual errors.

From a biological perspective, the observed correlations indicate that M-polynomial indices successfully capture underlying structural features that influence drug-relevant behavior. This highlights their potential as reliable and cost-effective surrogates for experimental ADME profiling, especially in early-stage drug design involving polycyclic and polysaccharide-based therapeutics.

In summary, this work underscores the practical significance of M-polynomial indices as robust and interpretable descriptors in cheminformatics, providing a foundation for future studies in computational drug discovery and molecular modeling.

## Data Availability

All data generated or analyzed during this study are included in this published article.
